# Effect of Vitamin D Supplementation on Bone Mass in Infants With 25-Hydroxyvitamin D Concentrations Less Than 50 nmol/L: A Prespecified Secondary Analysis of a Randomized Clinical Trial

**DOI:** 10.1001/jamapediatrics.2022.5837

**Published:** 2023-02-13

**Authors:** Nathalie Gharibeh, Maryam Razaghi, Catherine A. Vanstone, Olusola F. Sotunde, Laura Glenn, Kristina Mullahoo, Zahra Farahnak, Ali Khamessan, Shu Qin Wei, Dayre McNally, Frank Rauch, Glenville Jones, Martin Kaufmann, Hope A. Weiler

**Affiliations:** 1 School of Human Nutrition, McGill University, Sainte-Anne-de-Bellevue, Quebec, Canada; 2 Department of Biochemistry, Memorial University of Newfoundland, St John’s, Newfoundland and Labrador, Canada; 3 Europharm International Canada Inc, Montreal, Quebec, Canada; 4 Institut National de Santé Publique du Québec, Montreal, Quebec, Canada; 5 Department of Pediatrics, Children’s Hospital of Eastern Ontario, University of Ottawa, Ottawa, Ontario, Canada; 6 Shriners Hospital for Children, Montreal, Quebec, Canada; 7 Department of Biomedical and Molecular Sciences, Queen’s University, Kingston, Ontario, Canada; 8 Nutrition Research Division, Bureau of Nutritional Sciences, Health Products and Food Branch, Health Canada, Ottawa, Ontario, Canada

## Abstract

**Importance:**

The dose of supplemental vitamin D needed in infants born with serum 25-hydroxyvitamin D (25[OH]D) concentrations less than 50 nmol/L (ie, 20 ng/mL) is unclear.

**Objective:**

To determine whether a higher dose (1000 IU vs 400 IU per day) is required in infants born with 25(OH)D concentrations less than 50 nmol/L for bone mineral accretion across infancy.

**Design, Setting, and Participants:**

In this prespecified secondary analysis of a double-blinded randomized clinical trial, conducted from March 2016 to March 2019 in a single center in Greater Montreal, Quebec, Canada, a consecutive sample of 139 healthy term singletons were recruited from 866 infants screened for vitamin D status at birth. Data were analyzed from June 2021 to November 2022.

**Interventions:**

Capillary blood was collected 24 to 36 hours after birth to measure serum total 25(OH)D concentrations. Infants with 25(OH)D concentrations less than 50 nmol/L were randomized to receive either 1000 IU or 400 IU per day of oral vitamin D_3_ supplementation from age 1 to 12 months. Infants with 25(OH)D concentrations of 50 nmol/L or greater formed a reference group.

**Main Outcomes and Measures:**

Measures at age 1, 3, 6, and 12 months were preplanned and included whole-body bone mineral content, lumbar spine bone mineral content, and bone mineral density using dual-energy x-ray absorptiometry, and serum 25(OH)D_3_ using liquid chromatography tandem mass spectrometry.

**Results:**

Of 139 included infants, 81 (58.3%) were male, and the median (IQR) gestational age at birth was 39.6 (38.9-40.6) weeks. A total of 49 infants were included in the 1000 IU per day group, 49 infants in the 400 IU per day group, and 41 in the reference group. Mean (SD) whole-body bone mineral content was not different between trial groups over time (1000 IU per day, 173.09 [2.36] g; 400 IU per day, 165.94 [66.08] g). Similarly, no differences were observed in lumbar spine bone mineral content or density. Mean (SD) serum 25(OH)D_3_ concentrations were significantly higher in the 1000 IU per day group from age 3 to 12 months (3 months, 115.2 [35.3] nmol/L; 6 months, 121.6 [34.4] nmol/L; 12 months, 99.6 [28.8] nmol/L) compared with the 400 IU per day trial group (3 months, 77.4 [23.3] nmol/L; 6 months, 85.1 [18.6] nmol/L; 12 months, 82.3 [14.3] nmol/L).

**Conclusions and Relevance:**

In this study, a higher dose of vitamin D supplementation in infants born with 25(OH)D concentrations less than 50 nmol/L did not present advantages to bone mass in infancy. This study supports a standard dose of 400 IU per day of vitamin D supplementation for breastfed infants in Montreal.

**Trial Registration:**

ClinicalTrials.gov Identifier: NCT02563015

## Key Points

**Question:**

Does a dosage of 1000 IU per day compared with 400 IU per day of supplemental vitamin D in infants born with serum 25-hydroxyvitamin D concentrations less than 50 nmol/L (ie, 20 ng/mL) present advantages to bone outcomes throughout infancy?

**Findings:**

In this prespecified secondary analysis of a double-blinded randomized clinical trial including 139 healthy term infants, whole-body bone mineral content, lumbar spine bone mineral content and density, and bone biomarkers were not different among dosage groups from age 1 to 12 months.

**Meaning:**

This study supports a standard daily supplemental dose of 400 IU of vitamin D in breastfed infants in Montreal, even if born with serum 25-hydroxyvitamin D concentrations less than 50 nmol/L.

## Teaser

This prespecified secondary analysis of a randomized clinical trial evaluates whether a higher dose of supplemental vitamin D (1000 vs 400 IU per day) is required in infants born with 25-hydroxyvitamin D (25[OH]D) concentrations less than 50 nmol/L for bone mineral accretion across infancy.

## Introduction

Vitamin D status at birth reflects maternal-fetal transfer of 25-hydroxyvitamin D (25[OH]D).^1^ The Recommended Dietary Allowance for vitamin D for pregnancy and lactation is set at 600 IU per day.^2^ When the expecting mother has 25(OH)D concentrations less than 50 nmol/L (ie, 20 ng/mL), infants are born with elevated risk of vitamin D insufficiency (25[OH]D less than 50 nmol/L) or are deficient (25[OH]D less than 30 nmol/L).^3^ Human milk does not provide vitamin D in amounts consistent with the Adequate Intake for infants (400 IU per day).^4^ Therefore, public health policies in North America recommend that all breastfed infants begin vitamin D supplementation (400 IU per day) shortly after birth.^5,6^ The recommended amount of vitamin D for infants is set in accordance with intakes that maintain serum 25(OH)D concentration, the best marker of vitamin D status, in the range of 40 to 50 nmol/L in support of bone health.^2^ In Canada, based on studies from different provinces, the proportion of infants with insufficient vitamin D status ranges from 24.4% in Quebec City (cord serum 25[OH]D concentration less than 50 nmol/L) to 28% in Calgary and Edmonton (cord serum 25[OH]D concentrations less than 50 nmol/L) and 36% in Winnipeg (cord serum 25[OH]D concentrations less than 27.5 nmol/L).^7^

A dose-response relation exists between vitamin D intake and 25(OH)D concentration in infants, and the lower the initial concentration, the greater the rise in vitamin D status.^8,9^ Adherence to 400 IU per day of vitamin D supplementation during the first year of life prevents vitamin D deficiency rickets.^10^ Trials of dosages more than 400 IU per day conducted in infants with sufficient vitamin D status show that bone mineral content (BMC) and bone mineral density (BMD) are not affected.^11,12^ In another trial in infants with 25(OH)D concentrations less than 50 nmol/L at baseline, supplementation (200 to 800 IU/d) from age 2 to 9 months elevated 25(OH)D more than 50 nmol/L, and bone mass was not different in a small subgroup analysis.^13^ It remains unclear whether BMC is compromised in infants born with serum 25(OH)D concentrations less than 50 nmol/L and whether a higher dose of vitamin D supplementation is required to build vitamin D stores and to support bone health across infancy.

The objective of this study was to compare the effect of 1000 IU per day of oral vitamin D supplementation vs 400 IU per day on bone health from age 1 to 12 months in infants born with serum 25(OH)D concentrations less than 50 nmol/L and whose mothers had an intent to breastfeed for at least 3 months. Our hypothesis was that infants born with serum 25(OH)D concentrations less than 50 nmol/L and provided with a supplement of 400 IU per day (compared with 1000 IU per day) would have lower bone mineral accretion by 3 months without resolution at age 12 months.

## Methods

### Study Design

**Figure 1.**
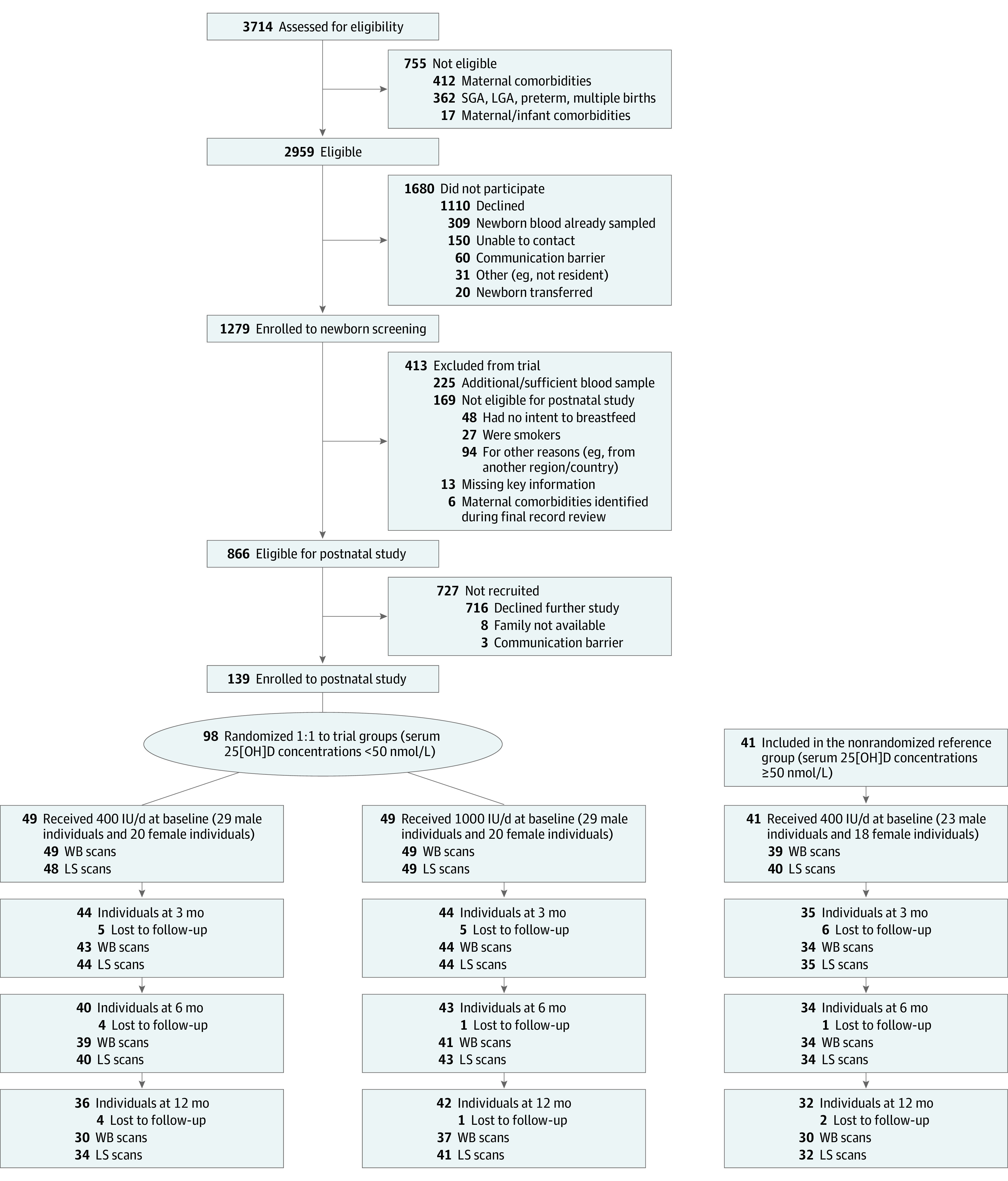
Participant Flow Diagram Participant flow diagram showing number of mother-infant dyads assessed for eligibility 24 to 36 hours after delivery, enrolled to newborn screening, screened, enrolled to the postnatal study, and allocated to either the trial group (serum 25-hydroxyvitamin D (25[OH]D) concentrations less than 50 nmol/L [ie, 20 ng/mL]) or reference group (serum 25[OH]D concentrations of 50 nmol/L or more). Infants allocated to the trial group were randomized to receive either 400 or 1000 IU per day. Infants in the reference group received 400 IU per day. Sample size per group at each time point reflects the number analyzed for biomarkers and the number of whole-body (WB) and lumbar spine (LS) scans available at each study visit are reported. LGA indicates large for gestational age; SGA, small for gestational age.

This was a prespecified secondary analysis of a double-blinded, parallel group randomized clinical parallel group trial conducted in Montreal, Quebec, Canada, from March 2016 to March 2019 and followed the Consolidated Standards of Reporting Trials (CONSORT) reporting guideline. The trial protocol can be found in Supplement 1. The trial was designed with lean body mass as the primary outcome, as reported in detail elsewhere,^14^ with assessment of bone mass among the a priori secondary outcomes. In brief, healthy term singleton infants at an appropriate weight for gestational age and whose mothers had an intent to breastfeed to at least age 3 months were recruited at birth. Infants with serum 25(OH)D concentrations less than 50 nmol/L at birth were randomized at age 1 month (baseline) to one of 2 trial groups, and those with birth serum 25(OH)D concentrations of 50 nmol/L or greater (sufficient vitamin D status) formed a reference group. Infants in the trial groups were randomized 1:1 to receive either 400 IU or 1000 IU per day of oral vitamin D_3_ supplementation from age 1 to 12 months. Details of the allocation and supplements have been reported along with data showing that growth (weight, length, and head circumference) was maintained within normal ranges across the study according to age-sex *z* scores generated using the World Health Organization growth standard.^14^ Infants in the reference group received 400 IU per day and served as a reference group for bone health outcomes. Infants were assessed at age 1 (baseline), 3, 6, and 12 months (Figure 1). Mothers self-reported their own race, which was subsequently categorized as Black, East/Southeast Asian, Latino, Middle Eastern, South Asian, White, or other, as described in detail elsewhere.^14^

Ethical approval was obtained from St. Mary’s Hospital Research Ethics Committee, which oversees research ethics for the Lakeshore General Hospital (SMHC-15-34), where newborn recruitment took place. The study was also reviewed and approved by Health Canada Research Ethics Board (REB 2019-033H) and Privacy Management Division (HC-PR-2019-000024). Written consent was obtained from the parents at the newborn screening and the baseline visit. The trial was conducted at the Mary Emily Clinical Nutrition Research Unit, McGill University, Sainte-Anne-de-Bellevue, Quebec, Canada.

### Bone Outcomes

Whole-body (WB) and lumbar spine (LS) vertebrae L1 to L4 BMC and BMD were measured using dual-energy x-ray absorptiometry methodology, as described elsewhere.^11^ WB BMC and LS BMC accretion rates (age 1 to 3 months, 3 to 6 months, and 6 to 12 months) were calculated as change in BMC/change in age. Coefficient of variation for each of BMC, BMD, and bone area were less than 1% based on Hologic spine phantom No. 14774.

### Biochemistry

Blood samples from the infants and their mothers were collected, as previously reported.^14^ Spot urine samples were collected from the infant using a pediatric urine bag at each visit and stored at −80 °C until analyzed for minerals and bone resorption biomarkers.

In infants at birth, serum total 25(OH)D concentrations were measured using an automated chemiluminescent immunoassay (LIAISON analyzer; DiaSorin), as previously reported,^14^ and standardized to the National Institute of Standards and Technology (NIST) reference materials,^15^ using Deming regression (standardized concentration [in nmol/L] calculated as 0.9634 [measured concentration] + 3.122).^16^

During the trial, infant serum (200 μL) was used to measure 25(OH)D_3_, 25(OH)D_2,_ 24,25-dihydroxyvitamin D_3_ (24,25[OH]_2_D_3_), 1,25-dihydroxyvitamin D_3_ (1,25[OH]_2_D_3_), and 1,24,25-trihydroxyvitamin D_3_ (1,24,25[OH]_3_D_3_) concentrations using liquid chromatography–mass spectrometry/mass spectrometry at Queen’s University according to published methods.^17,18^ Total 25(OH)D concentration was calculated as 25(OH)D_3_ + 25(OH)D_2._ Maternal total 25(OH)D concentration was measured in serum collected at the baseline visit. The laboratory participated in the Vitamin D Standardization-Certification Program and the Vitamin D External Quality Assessment Scheme and obtained certificates of proficiency. Accuracy was within 5% of the NIST standard reference materials, with interassay coefficient of variation less than 10%.

Plasma procollagen type 1 N-terminal propeptide (P1NP; Human P1NP ELISA; Creative Diagnostics) and urinary alpha telopeptide of type 1 collagen (CTX-I; EIA; Immunodiagnostic Systems) were measured as recommended^19^ as well as parathyroid hormone (PTH; Human PTH 1-84 EIA; Quidel; MicroVue) using immunoassays with an interassay coefficient of variation less than 10%. Urinary creatinine concentration, calcium to creatinine ratio, and phosphate to creatinine ratio were measured using an autoanalyzer (UniCel DxC600; Beckman Coulter) at McGill University Health Centre Clinical Chemistry Laboratory, certified by the International Organization for Standardization. Urinary CTX-I to creatinine ratios were calculated. Infant blood-ionized calcium (iCa) was measured in whole blood (65 μL) using a blood gas analyzer (ABL80 FLEX; Radiometer Medical), calibrated daily.

### Dietary and Lifestyle Data

At baseline, maternal nutritional intake (energy, protein, carbohydrates, fat, vitamin D, calcium, magnesium, and phosphorus) during pregnancy from food and supplements was assessed using a validated semiquantitative food frequency questionnaire,^20^ and demographic and lifestyle information were surveyed. In addition, constitutive skin pigmentation of the infants was measured at the inner upper arm, as reported elsewhere.^14^ At each study visit, breastfeeding status (yes [exclusive or mixed] or no [none]) was surveyed. Age of introduction of solid foods was surveyed at the age 6 months visit; dietary intake throughout the trial was assessed as previously reported and not different between trial groups.^14^

### Statistical Analysis

This analysis is the secondary objective of the trial. The primary objective was focused on lean mass outcomes, with the aim of recruiting a minimum of 46 infants per trial group and up to 74 to account for dropouts.^14^ Data are presented as means with SDs, medians with IQRs, or counts and percentages.

Differences between the trial groups over time in bone outcomes (dual-energy x-ray absorptiometry and biomarkers), vitamin D metabolites, and safety biomarkers (iCa, urinary calcium to creatinine ratio, and urinary phosphate to creatinine ratio) were tested using linear mixed-effects regression models with participant-level random intercepts and slopes for time. We used a first-order autoregressive covariance structure selected based on the correlation matrix and the lowest Akaike information criterion. The variables included group-by-time interaction, time, and participant number. Skin pigmentation, UV-B period and season at birth, infant sex, and socioeconomic and demographic characteristics were explored but not retained in the model. Data were not imputed given that the mixed-effects model used all available data, and data were assumed to be missing at random.^21^ The Akaike and Bayesian information criteria and R^2^ values were used to examine model fit. Tukey-Kramer tests were used for post hoc comparisons, adjusted for multiple comparisons. Normality of the residuals was tested using Shapiro-Wilk test. For all outcomes, no formal statistical comparison to the reference group was conducted. Differences in proportions of vitamin D–sufficient infants over time between the trial groups at 3, 6, and 12 months were tested using the proc glimmix function with CHISQ option; the variables were group-by-time interaction, time, and participant number. Differences in breastfeeding status and age of introduction of solid foods were tested using linear mixed-effects regression models (continuous variables) or χ^2^ or Fisher exact tests (categorical variables).

A post hoc analysis of covariance tested differences between the trial groups in bone outcomes (WB BMC, LS BMC, and LS BMD) with corresponding baseline values for each dependent variable tested as covariates. In these models, the repeated measures were at 3, 6, and 12 months.

All statistical analyses were conducted using SAS University Edition (SAS Institute), and statistical significance was set at *P* < .05 after adjustment for multiple comparisons. All *P* values were 2-sided.

## Results

Characteristics of infants and their mothers are provided in the Table and eTable 1 in Supplement 2.^22^ Of 139 included infants, 81 (58.3%) were male, and the median (IQR) gestational age at birth was 39.6 (38.9-40.6) weeks. A total of 49 infants were included in the 1000 IU per day group, 49 infants in the 400 IU per day group, and 41 in the reference group. Compliance to infant vitamin D supplementation was 85% or more overall. A total of 29 dropouts (20.9%) occurred; characteristics of those who completed the study compared with those who dropped out is reported elsewhere.^14^ Proportions of infants who were breastfed at each time point and age of introduction of solid foods are reported in eTable 1 in Supplement 2.

**Table.** Characteristics at Birth and at Baseline

Characteristic	Group, No. (%)		
	400 IU/d (n = 49)	1000 IU/d (n = 49)	Reference (n = 41)
**Infant characteristics**			
Sex			
Female	20 (40.8)	20 (40.8)	18 (43.9)
Male	29 (59.2)	29 (59.2)	23 (56.1)
Season at birth^a^			
Winter	10 (20.4)	15 (30.6)	11 (26.8)
Spring	20 (40.8)	10 (20.4)	9 (20.0)
Summer	8 (16.3)	12 (24.5)	12 (29.3)
Fall	11 (22.4)	12 (24.5)	9 (20.0)
Gestational age at birth, median (IQR), wk	39.7 (39.0-40.5)	39.6 (38.8-40.7)	39.6 (38.9-40.4)
Weight for age *z* score at birth, median (IQR)	0 (−0.5 to 0.7)	0.3 (−0.6 to 0.6)	0.4 (−0.1 to 0.8)
Total 25(OH)D at birth (standardized), nmol/L^b^	32.8 (8.9)	36.3 (11.5)	68.6 (12.8)
Total 25(OH)D at baseline, nmol/L^c^	45.8 (14.1)	46.4 (14.4)	60.9 (14.9)
25(OH)D_3_ at baseline, nmol/L^c^	44.3 (14.3)	44.7 (15.3)	59.5 (15.3)
Inner arm ITA at baseline, °	30.0 (19.8)	31.2 (18.1)	42.0 (8.6)
**Mother characteristics**			
Age at delivery, median (IQR), y	33.4 (29.8-36.4)	32.2 (27.9-34.4)	31.9 (28.7-35.8)
Gravida, median (IQR)	2 (1.5-3.0)	2 (1-2)	2 (1-3)
Vaginal birth	31 (63.3)	35 (71.4)	29 (70.7)
Gestational weight gain category^d^			
Inadequate	14 (28.6)	12 (25.0)	7(17.1)
Adequate	11 (22.5)	15 (31.3)	18 (43.9)
Excessive	24 (49.0)	21 (43.8)	16 (39.0)
Pregravid BMI, median (IQR)^e^	24.3 (21.2-27.1)	23.4 (21.9-27.8)	23.4 (21.3-24.7)
Postsecondary education	41 (83.7)	45 (91.8)	40 (97.6)
Race and ethnicity^f^			
Black	4 (8.2)	5 (10.2)	0
East/Southeast Asian	5 (10.2)	1 (2.0)	2 (4.9)
Latino	1 (2.0)	4 (8.2)	5 (12.2)
Middle Eastern	8 (16.2)	7 (14.3)	1 (2.4)
South Asian	3 (6.1)	3 (6.1)	0
White	22 (44.9)	24 (49.0)	31 (75.6)
Multiple races or unknown	6 (12.2)	5 (10.2)	2 (4.9)
Total 25(OH)D at baseline, nmol/L^c^	53.6 (14.1)	59.9 (22.1)	90.5 (20.4)
Family income per y, CAD$ (USD$)			
<50 000 (<37 338)	10 (20.4)	13 (26.5)	4 (9.8)
≥50 000-99 999 (≥37 338-74 677)	17 (34.7)	11 (22.4)	12 (29.3)
≥100 000 (≥74 677)	15 (30.6)	16 (30.7)	20 (48.8)
Not reported	7 (14.3)	9 (18.4)	5 (12.2)

Abbreviations: BMI, body mass index; ITA, individual typology angle; 24,25(OH)_2_D_3_, 24,25-dihydroxyvitamin D_3_; 25(OH)D, 25-hydroxyvitamin D; 25(OH)D_3_, 25-hydroxyvitamin D_3_.

^a^ Seasons are based on equinox and solstice dates for each year.

^b^ Serum 25(OH)D concentrations measured using chemiluminescent immunoassay and standardized using Deming regression (standardized concentration [in nmol/L] calculated as 0.9634 [measured concentration] + 3.122).^16^

^c^ Measured using liquid chromatography–mass spectrometry/mass spectrometry.

^d^ Gestational weight gain categories were classified according to pregravid BMI using the Institute of Medicine classification.^22^

^e^ Calculated as weight in kilograms divided by height in meters squared.

^f^ Mothers self-reported their own race, as described in detail elsewhere.^14^

**Figure 2.**
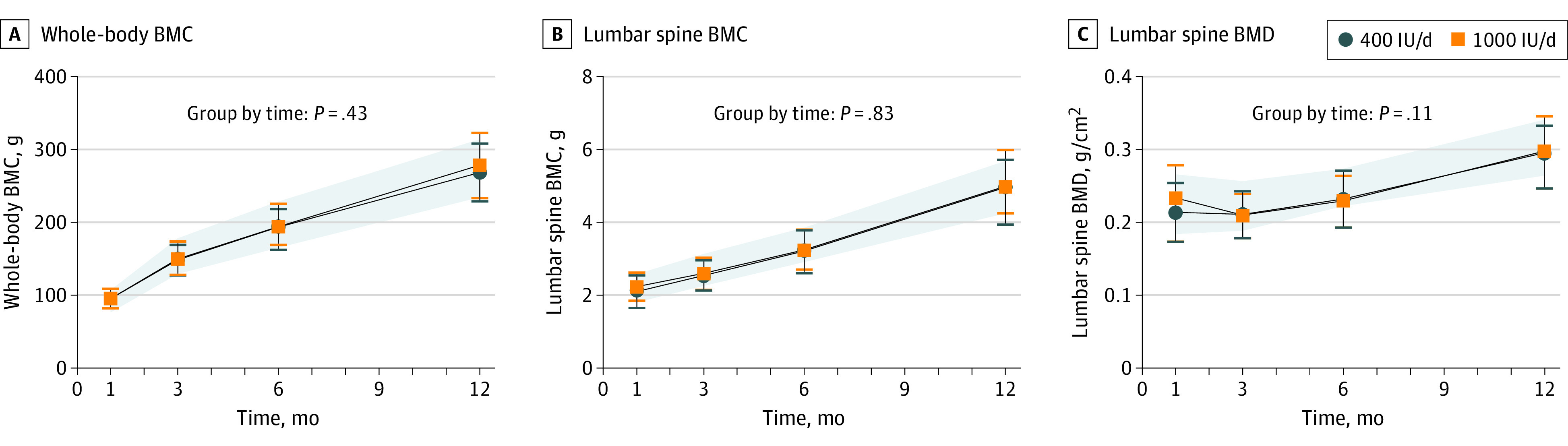
Whole-Body Bone Mineral Content (BMC), Lumbar Spine BMC, and Lumbar Spine Bone Mineral Density (BMD) of Infant Groups Over Time Data are reported as means with SDs and were analyzed using a linear mixed-effects regression model for group-by-time interaction and time; the model included participant-level random intercepts and slopes for time. Post hoc testing showed no significant differences between trial groups over time adjusted for multiple comparisons except for time, which was significant for all comparisons. A total of 469 whole-body scans and 484 lumbar spine scans were obtained of a possible 489 across all time points. The remaining were not obtained or analyzed due to movement artifacts. The shaded areas correspond to the reference group SDs.

WB BMC, WB BMC per kilogram bodyweight or BMC per centimeter, LS BMC, and LS BMD were not different between groups across the trial (Figure 2; eTable 2 in Supplement 2). WB BMC and LS BMC accretion rates (grams per month) did not differ between groups over time (eFigure 1 in Supplement 2). No differences were observed between the trial groups in bone outcomes (WB BMC, LS BMC, and LS BMD) when including baseline values as a covariate.

**Figure 3.**
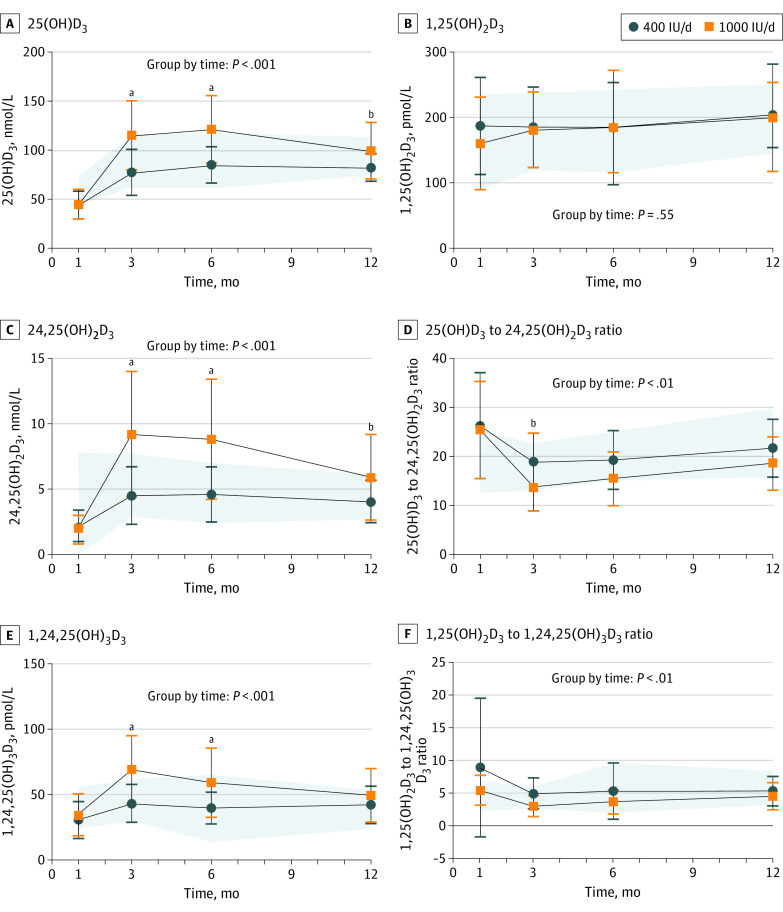
Serum Concentrations of 25(OH)D_3_, 1,25(OH)_2_D_3_, 24,25(OH)_2_D_3_, 25(OH)D_3_ to 24,25(OH)_2_D_3_ Ratio, 1,24,25(OH)_3_D_3_, and 1,25(OH)_2_D_3_ to 1,24,25(OH)_3_D_3_ Ratio of Infant Groups Over Time Data are reported as means with SDs and were analyzed using a linear mixed-effects regression model for group-by-time interaction and time; the model included participant-level random intercepts and slopes for time. Post hoc testing showed differences between trial groups over time for the 1000 IU per day group vs 400 IU per day group were adjusted for multiple comparisons except for time, which was significant for all comparisons. The shaded areas correspond to the reference group SDs. 25(OH)D_3_ indicates 25-hydroxyvitamin D_3_; 1,25(OH)_2_D_3_, 1,25-dihydroxyvitamin D_3_; 24,25(OH)_2_D_3_, 24,25-dihydroxyvitamin D_3_; 1,24,25(OH)_3_D_3_, 1,24,25-trihydroxyvitamin D_3_. ^a^*P* < .001. ^b^*P* < .05.

Serum 25(OH)D_3_ and 24,25(OH)_2_D_3_ concentrations in the 1000 IU per day group were higher at age 3, 6, and 12 months compared with the 400 IU per day trial group (Figure 3; eTable 3 in Supplement 2). By design, the proportion of infants with sufficient vitamin D status at baseline ranged from 38% to 45% in the trial groups; thereafter, proportions were not different at 3-, 6-, and 12-month time points (eFigure 2 in Supplement 2). The ratio of 25(OH)D_3_ to 24,25(OH)_2_D_3_ was higher in the 1000 IU per day group at 3 months but not at 6 or 12 months. Calcitriol concentrations (Figure 3; eTable 3 in Supplement 2) were not different between trial groups over time. Serum 1,24,25(OH)_3_D_3_ concentration was higher in the 1000 IU per day compared with the 400 IU per day group at 3 and 6 months, with no difference in the ratio of 1,25(OH)_2_D_3_ to 1,24,25(OH)_3_D_3_. Serum 3-epi-25(OH)D_3_ concentrations were higher in the 1000 IU per day group compared with the 400 IU per day trial group at age 3 and 6 months but not at 12 months (eTable 3 in Supplement 2).

**Figure 4.**
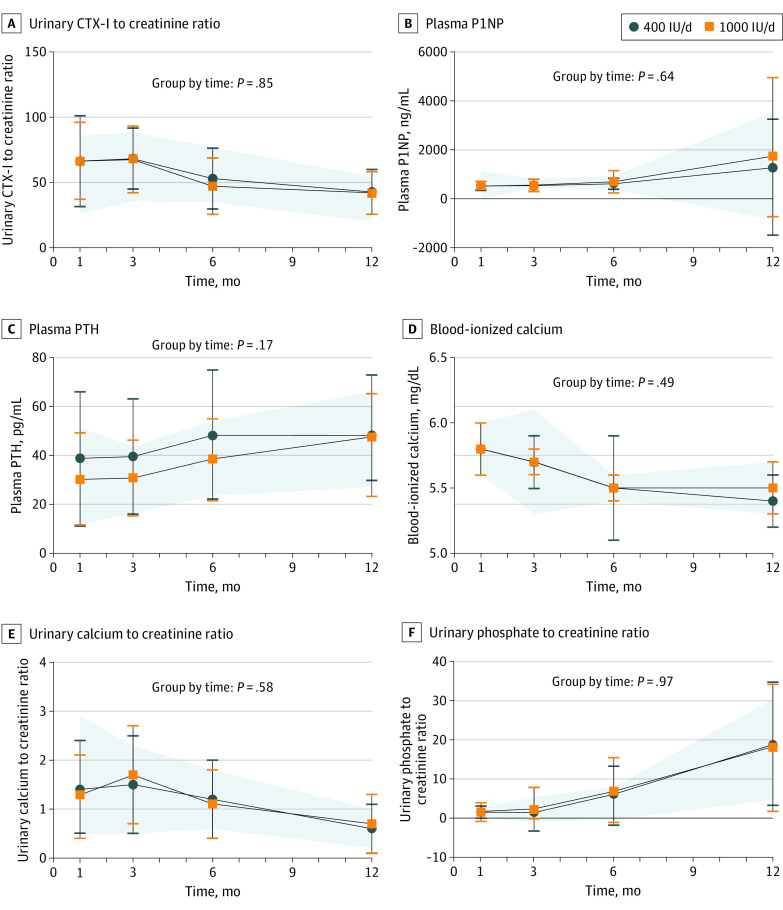
Biomarkers of Calcium and Bone Metabolism of Infant Groups Over Time Data are reported as means with SDs and were analyzed using a linear mixed-effects regression model for group-by-time interaction and time; the model included participant-level random intercepts and slopes for time. Post hoc testing showed no differences between trial groups over time. The 1000 IU per day group and 400 IU per day group were adjusted for multiple comparisons except for time, which was significant for all comparisons. The shaded areas correspond to the reference group SDs. CTX-I indicates urinary alpha telopeptide of type 1 collagen; P1NP, procollagen type 1 N-terminal propeptide; PTH, parathyroid hormone.

Biomarkers of bone formation and resorption, iCa, PTH, and urinary calcium to creatinine and phosphate to creatinine ratios were not different between the groups over time (Figure 4; eTable 4 in Supplement 2).

## Discussion

In the absence of robust trials investigating the effect of a dose of vitamin D supplementation higher than the standard of care (400 IU per day) on bone mineral accretion and density in infants born with 25(OH)D concentrations less than 50 nmol/L, this prespecified secondary analysis of a randomized clinical trial provides information to help guide recommendations for vitamin D supplementation in this understudied population. Infants at elevated risk of insufficient vitamin D status provided with a daily supplement of 400 IU (compared with 1000 IU per day) did not have compromised bone outcomes (WB BMC, LS BMC, or LS BMD) across infancy. This is in line with findings from a study by Ziegler et al,^13^ in which doses of vitamin D supplementation ranging from 200 to 800 IU per day did not impact WB BMC from age 2 to 9 months in a small subsample (n = 13 at 9 months) of exclusively breastfed infants with 25(OH)D concentrations less than 50 nmol/L at baseline. Similarly, Gallo et al^11^ and Rosendahl et al^12^ investigated BMC and BMD in predominantly vitamin D–sufficient infants and showed no differences according to dose of vitamin D supplementation ranging from 400 to 1600 IU per day. The consistent absence of a dose-response in bone mass suggests a saturable effect in bone mineral accretion when vitamin D is supplemented at 400 IU per day.

The results of this study and others^11,12,13^ complement a recent Cochrane review comparing 400 IU per day of supplemental vitamin D to placebo that reported no difference in BMC of the distal radius based on 2 trials in breastfed infants, whereas the supplement was protective against serum 25(OH)D less than 50 nmol/L in 4 other trials.^23^ More recently, a trial in newborn infants with serum 25(OH)D concentrations less than 50 nmol/L confirmed in most participants (67%) that 400 IU per day compared with placebo had no effect on BMD of the LS measured at age 4 months.^24^ This was observed even though the placebo group demonstrated declines in serum 25(OH)D concentrations, and those with concentrations less than 25 nmol/L at age 4 months had lower BMC and higher PTH. It is thus prudent to provide the breastfed infant with protection against vitamin D deficiency as soon as possible after birth using a vitamin D supplement of 400 IU per day, as is recommended in North America.^5,6^

In accordance with no evidence of a dose-response in bone mass with vitamin D supplementation and achievement of sufficient vitamin D status, no differences in biomarkers of bone formation or resorption due to vitamin D dose were noted in our trial from age 1 to 12 months. These markers lack reference data in infancy, and efforts to standardize biomarkers of bone metabolism are needed.^19^ During infancy, P1NP is higher than in childhood and adolescence, indicative of the rapid bone modeling for linear growth during the first year of life.^25,26^ Bone biomarkers are dynamic with a relatively short half-life and therefore reflect acute changes in physiology. Consistent findings on both biomarkers of bone metabolism and bone mineral accretion rates show no differences among groups and confirm that in healthy term-born infants, 400 IU per day is enough to support bone health.

Serum 25(OH)D_3_, 24,25(OH)_2_D_3_, and 3-epi-25(OH)D_3_ concentrations were different between our trial groups. In conditions of low vitamin D status, there is a decreased activity of the 24-hydroxylase enzyme (CYP24A1).^27^ This helps to explain why the ratio of 25(OH)D_3_ to 24,25(OH)_2_D_3_ were not different between the trial groups at 6 or 12 months, as both groups started the trial with 25(OH)D concentrations less than 50 nmol/L on average, and by age 3 months were replete in vitamin D. To our knowledge, the only other dose-response trial reporting on different vitamin D metabolites in infancy also reported that higher doses of vitamin D supplementation resulted in greater concentrations of different vitamin D metabolites, including 3-epi-25(OH)D_3_ and 24,25(OH)_2_D_3_ that vary over time.^11^

The lack of dose-response of 1,25(OH)_2_D_3_ to supplemental dose is consistent with tightly regulated concentrations. Concentrations of 1,25(OH)_2_D_3_ eventually decrease when the precursor 25(OH)D concentrations are less than 25 nmol/L,^28^ as observed in only 6 infants with serum 1,25(OH)_2_D_3_ concentrations ranging from 129.5 to 337.9 pmol/L at baseline. Interestingly, our novel data on 1,24,25(OH)_3_D_3_ and the molar ratio of 1,25(OH)_2_D_3_ to 1,24,25(OH)_3_D_3_ suggest that 1,25(OH)_2_D_3_ is catabolized as evidenced by the rise in 1,24,25(OH)_3_D_3_ at age 3 and 6 months in the 1000 IU per day group with no difference in the ratio. The relatively high 1,25(OH)_2_D_3_ concentrations and modest increases in PTH observed across infancy^29^ more likely relate to the high calcium demand in rapidly growing infants before 6 months. The Adequate Intake for calcium is 200 mg from birth to age 6 months and 260 mg at age 7 to 12 months.^2^ The reduced consumption of breastmilk and weaning after age 6 months would increase calcitriol concentrations given the greater bioavailability of calcium in breastmilk compared with solid foods.^30^ The decrease in iCa observed over the 12 months may reflect maturation of liver function and higher circulating albumin concentrations, which bind calcium.^29^ Nonetheless, the lack of differences among groups in iCa confirms that calcium homeostasis is maintained in infancy, even when serum 25(OH)D concentrations are less than 40 to 50 nmol/L, the desirable concentrations in infancy according to which the Adequate Intake was defined.^2^ Given that iCa and calcitriol are the principal determinants of PTH concentration, it is not surprising that no differences were observed in PTH concentrations among our study groups or among those of other trials,^13,24^ provided sufficient vitamin D status is achieved.

### Strengths and Limitations

Strengths of this study are its design that implemented targeted entrance criteria on the basis of serum 25(OH)D concentrations less than 50 nmol/L at birth and that it provides valuable data on bone mass, calcium homeostasis, and multiple vitamin D metabolites in infancy. This study also has limitations. Outcomes were measured using criterion standards for assessment of bone mass and vitamin D status.^15,31^ Based on maternal self-report, 44.6% identified as a racial and ethnic minority group. Our results are therefore generalizable to visible minority groups, which constitute 22.3% of the Canadian population and thus may have been overrepresented in our sample.^32^ It would have been of added value to have vitamin D metabolites measured at birth using liquid chromatography–mass spectrometry/mass spectrometry, as the immunoassay used may have underestimated 25(OH)D concentrations.^33^ Nonetheless, the bias is unlikely to be clinically meaningful as shown by the standardization of the 25(OH)D concentrations to the NIST reference materials.^15^ Other limitations include the attrition rate (20.9%), which was often due to the busy schedules of the parents.

## Conclusions

In conclusion, in infants with 25(OH)D concentrations less than 50 nmol/L at birth, both 400 and 1000 IU per day of vitamin D supplementation normalized and maintained 25(OH)D concentrations that align with skeletal health. The 1000 IU per day dosage of vitamin D supplementation did not lead to measurable improvements in bone health outcomes. Evidence from this Montreal-based study suggests that the standard of care of 400 IU per day is enough to support bone health of breastfed infants born with serum 25(OH)D concentrations less than 50 nmol/L.

## Notes

Supplement 1. Trial Protocol

Supplement 2. **eTable 1.** Infant Breastfeeding Status and Age of Introduction of Solid Foods and Maternal Nutrient Intake From Food and Supplements During Pregnancy by Group **eTable 2.** Whole Body and Lumbar Spine Bone Mass of Infants by Groups Over Time **eTable 3.** Serum Vitamin D Metabolites of Infants by Groups Over Time **eTable 4.** Biomarkers of Calcium and Bone Metabolism of Infants by Groups Over Time **eFigure 1.** Whole Body BMC and Lumbar Spine BMC Accretion Rates of infant Groups Over Time **eFigure 2.** Proportions of Vitamin D Sufficient Infants by Groups Over Time

Supplement 3. Data Sharing Statement
