# Supplementary material for: Effect of Vitamin D Supplementation on Bone Mass in Infants With 25-Hydroxyvitamin D Concentrations Less Than 50 nmol/L: A Prespecified Secondary Analysis of a Randomized Clinical Trial
Source: JAMA Pediatr. 2023 Feb 13;177(4):353–62. doi: 10.1001/jamapediatrics.2022.5837 (PMC9926359; doi:10.1001/jamapediatrics.2022.5837)
Supplement: Supplement 1. — Trial Protocol [file jamapediatr-e225837-s001.pdf]

Can Correction of Low Vitamin D Status in Infancy Program for a Leaner Body Composition?

Version: 1.1[1]

Date of Extraction: March 18, 2022

ClinicalTrials.gov Identifier: NCT02563015
Sponsor: McGill University
Principal investigator: Dr. Hope Weiler
Team of co-applicants: Dr. Katherine Gray Donald, Dr. Frank Rauch, Dr. Dayre McNally, Dr. Shu Qin Wei, Dr. Glenville Jones, Dr. Sarah Kimmins

[1] The protocol outlined in this report was extracted from the original operating grant as submitted to the Canadian Institutes of Health Research March 2, 2015. No changes were made to the protocol of the trial aside from reformatting of references and addition of the trial registration. Only the content pertinent to the measurement and analysis of infant bone health was extracted.

Table of Contents

1. Brief layperson summary ***4***

2. Trial objectives ***4***

3. Proposed trial ***4***

3.1 What is the proposed trial design? **4**

3.2 The planned trial interventions? **4**

3.3 Practical arrangements for allocation? **4**

3.4 What are the proposed methods for protecting against sources of bias? **5**

3.5 What are the inclusion/exclusion criteria? **5**

3.6 What is the proposed duration of treatment period? **5**

3.7 Frequency and duration of follow up **5**

3.8 Primary and secondary outcomes **5**

3.9 How will the outcome measures be measured at follow up? **5**

3.10 Will health service research issues be addressed? **7**

3.11 What is the proposed sample size and what is the justification? **7**

3.12 What is the planned recruitment rate? **8**

3.13 Are there likely to be any problems with compliance? **8**

3.14 What is the likely rate of loss to follow up? **8**

3.15 How many centers will be involved? **8**

3.16 What is the proposed type of analyses? **8**

3.17 What is the proposed frequency of analyses? **9**

3.17 Are there any planned subgroup analyses? **9**

4. Trial management ***9***

4.1 What are the arrangements for day to day management of the trial? **9**

4.2 Describe the trial steering committee and if relevant the data safety and monitoring committee **10**

4.3 Describe any risks to the safety of participants involved in the trial. **10**

Diagram 1: Randomization scheme ***11***

Diagram 2: Safety protocol - For use at inception 3, 6 and 12 months ***12***

Table 1. Summary of study measurements and timing ***13***

Table 2. Study time-lines (6 year grant October 1, 2015 to September 30, 2021) ***14***

Table 3. Normative values and threshold values used in safetymonitoring[1] ***15***

5. References ***16***

6. References for Safety Monitoring *17*

1. Brief layperson summary

One in four infants are born with low amounts of vitamin D stored in their body. This study is designed to test whether improving vitamin D status quickly after birth helps infants to build muscle and bone mass and to normalize growth. This is important since the investigators have noticed in previous work that infants with low vitamin D have higher body weight relative to body length later on and that those who develop very good stores quickly have a leaner body type. Therefore, in this study infants with low stores early after birth will be given either the regular amount of supplementation or a higher amount to more rapidly build up the vitamin stores in the body. Infants in both groups will be measured for muscle and fat mass and bone mineral content and density at standardized ages during the first year of life and into the toddler years. The information will inform health care professionals and parents of the importance of establishing good vitamin D stores early in life. Vitamin D supplementation is a modifiable factor that is already recommended for all term born infants. Knowing how much is needed in infants born with low stores has not been tested in a controlled manner in Canada.

2. Trial objectives

Primary Trial Objective: To test whether rapid correction of vitamin D status early in the neonatal period results in greater lean mass by 3 mo of age and sustained to 3 y. It is hypothesized that neonates with low vitamin D status and provided a vitamin D supplement of 400 IU/d will have lower accretion of lean mass by 3 mo of age and thereafter to 3 y compared to infants given 1000 IU/d.

Secondary Trial Objectives are designed *a priori* to test:
1) Whether correction of low vitamin D status early in infancy using 1000 IU/d of vitamin D normalizes body composition (lean, fat, bone mass) across infancy and to 3 y of age;

Tertiary Objective: outside of the trial, establish a healthy reference group with good neonatal vitamin D stores (>50 nmol/L 25(OH)D) and given the AI of 400 IU vitamin D/d with all of the same measurements as the trial since these are not all available (e.g. IGF-1, IGFBP3 using same assays). The reference group will be frequency matched to trial infants based on skin color of the infants and season.

3. Proposed trial

3.1 What is the proposed trial design?

Double blinded randomized parallel group trial.

3.2 The planned trial interventions?

Standard of care 400 IU vs. 1000 IU vitamin D/d from baseline (1 wk) to 12 mo in neonates with low vitamin D status; and a reference group given 400 IU vitamin D/d.

3.3 Practical arrangements for allocation?

We will use RANDOMIZE.net according to variable block sizes and stratification, 2 treatment arms and verification of entry criteria. Stratification will be on measured skin color of the infant (**Diagram 1**). We will track the reference group using this system.

3.4 What are the proposed methods for protecting against sources of bias?

Both study products will be blinded to participating families and all researchers. Euro-Pharm will provide these supplements *in kind* in identical containers and the supplement will be identical in color, taste, texture etc. Each bottle will have a unique code to enable tracing back to the product dosage in the rare situation of an adverse event without unblinding the study. Euro-Pharm will provide the blinding codes in sealed envelopes, a copy each for the PI and the medical team. The allocation will be at the end of the baseline visit and care-givers will be educated by a nurse on how to properly give the supplement.

3.5 What are the inclusion/exclusion criteria?

For **screening**, inclusion criteria is any healthy term born infant of appropriate weight for gestational age (AGA) based on a Canadian growth reference [1] and born to a generally healthy mother of any race. For the randomized trial, infants must have serum 25(OH)D <50 nmol/L; and for those in the reference group >50 nmol/L. For both the trial and reference groups other **inclusion criteria** are: born to mothers with otherwise healthy pregnancy free of medications that impact vitamin D metabolism (except vitamin/mineral supplements) or fetal growth and intent to breastfeed to at least 3 mo. For the trial we will not exclude on the basis of maternal pre-pregnancy BMI, but will document pregnancy weight gain and obstetrical history from the medical record. For the healthy reference group we will recruit from our screening pool only infants born to mothers of healthy weights (pre-pregnancy BMI 18.5-24.9 kg/m^2) and weight gain according to IOM criteria [2]. **Exclusion criteria** are maternal smoking in pregnancy as it limits growth; and diabetes, preeclampsia, celiac disease, inflammatory bowel disease etc. and medications that impact vitamin D/mineral metabolism. All maternal medications will be documented.

3.6 What is the proposed duration of treatment period?

Trial is 1 wk to 12 mo of age; follow-up to 3 y.

3.7 Frequency and duration of follow up:

Baseline (1 wk), 3, 6 and 12 mo for the trial; then 2 and 3 y.

3.8 Primary and secondary outcomes:

Primary outcome: lean mass during the trial; with follow-up thereafter to 3 y. Secondary outcomes: body composition including bone mass and BMI z-scores, tissue accretion rates across the study period and follow-up; vitamin D status and mechanistic explanations (IGF-1).

3.9 How will the outcome measures be measured at follow up?

The study measurements and time-lines are shown in **Tables 1-2.** At baseline (1 wk), 3, 6, 12, 24 and 36 mo of age, infants will be seen at our research unit for measurement of anthropometry, body composition and bone as well as blood sampling, skin pigmentation and surveys for diet, activity and demographic information.

Anthropometry - All measurements in infancy will be obtained nude and for 24 to 36 mo the child wearing standardized light clothing, dry diaper and no shoes. Weight will be taken using an electronic scale with a dynamic weighing program (Mettler-Toledo Inc., Switzerland). Length (0.1 cm) will be measured using an infantometer until 24 mo of age (O'Learly Length Boards, Ellard Instrumentation Ltd., US) and height at 36 mo will be measured using a stadiometer (Seca Medical Scales and Measuring Systems, US). Head circumference will be measured (0.1 cm) using a non-stretchable tape (Perspective Enterprises, US) to complete the anthropometric panel. Weight-for-age, height-for-age, and BMI-for-age Z-sores will be calculated using WHO software (WHO AnthroPlus, Switzerland).

Body Composition Measurements - Body composition will be assessed using a fan-beam DXA (APEX version 13.3:3, Hologic 4500A Discovery Series, Bedford, MA). Each infant will wear a light sleeper with no metal or plastic components and a diaper and be scanned using the infant whole body software; at 24 and 36 mo standardized light clothing will be worn and scans captured using whole body software. Whole body scans provide lean mass (g and % of weight), fat mass (g and %), BMC and BMD. Lumbar vertebra 1-4 BMC and BMD will be captured according to guidelines [2].

Biochemistry Measurements - Capillary blood samples (0.5 ml) will be collected at screening; but venous sampling (1.0 ml: yields ~500-600 µl serum) used thereafter; samples will be centrifuged (2235 x g for 20 min at 4°C) to obtain serum (for biochemistry) and buffy coat (white blood cells for DNA) and stored frozen at -80°C until analysis. One 5 ml sample will be taken from parents at baseline (fasting) for measurement of serum 25(OH)D and buffy coat saved for future epigenetic work. For infant screening and maternal serum, total 25(OH)D will be measured using a dedicated auto-analyzer in the PI's laboratory (25 µl; 150 µl "dead volume" that is recovered, Liaison Diasorin Inc.); this assay will also be used for safety assessments at 3 and 6 mo, but is not to be used in analyzing the trial data outcomes as it does not capture all of the metabolites. Liquid chromatography tandem mass spectrometry (LC-MS/MS by Dr. Jones', Queen's Univ.) will be used to measure of $25(OH)D_3$, $3\text{-epi-}25(OH)D_3$, and $24,25(OH)_2D$ for all time-points from baseline to 36 mo. Briefly, 100 µl of serum will be used for these assays according to our methods [3, 4]. In addition, $1,25(OH)_2D$ (100 µl serum) will be similarly measured using an adapted LC-MS/MS method [5]. Both laboratories are certificated by the Vitamin D External Quality Assessment Scheme and will continue to participate in the National Institute of Standards and Technology quality assurance program. Blood-ionized calcium will be measured immediately using our portable unit (ABL80 FLEX Radiometer Medical A/S, Denmark) and compared to published standards [6]. Remaining sample will be used for IGF-1 (20 µl) using Liaison (Diasorin Inc.); PTH (25 µl; Immutopics Inc CAT#60-3100) and IGFBP3 (20 µl; R&D Systems CAT#SGB300) will be measured by ELISA. Sample for IGFBP3 will be pre-treated with protease inhibitors prior to storage. Plasma total calcium and phosphate (150 µl) and urinary calcium and phosphate:creatinine will be measured in a spot sample collected at each visit during the trial; Beckman Coulter UniCel DxC600 autoanalyzer as we describe [6]. We will reserve remaining sample for later measurement of C-telepeptide, propeptide of type 1 collagen (P1NP) as biomarkers related to bone.

Demographic, dietary and activity survey. At baseline, parents will be asked to complete a demographic survey regarding their anthropometry, ethnicity and race, income and education

using the same descriptors as defined by Statistics Canada. Infant dietary intake over the study period will be assessed using 3-day diet records completed by parents after each visit as described in detail [3]. While infants are breastfed, milk intake will be assessed by test-weighing of the infant before and after breast feeding for a 24-hour period [7] using a portable electronic scale (Tanita Corporation Inc., US). This will provide nutrient intakes to help explain growth. Dietary intake from other foods is documented using household measurement items and recorded on the 3-day record. All nutrient analysis will be completed using the Nutritionist Pro software version 4.7.0 (Axxya Systems LLC, Stafford, TX) and the most recent Canadian Nutrient File database (Health Canada). At 2 and 3 y of age, the Habitual Activity Estimation Scale Questionnaire (HAES) will be completed by parents for a weekday (Tuesday, Wednesday, or Thursday) and weekend day (Saturday) over the past 2 weeks [8, 9]. Parents divide their child's day into 4 segments (wake-up to breakfast, breakfast to lunch, lunch to dinner, dinner to bedtime). For each time interval, the % time spent in each activity level is used to estimate overall level of physical activity. The 4 activity levels as established by the HAES questionnaire are: "inactive" (lying down, sleeping, resting), "somewhat inactive" (sitting, watching television, activities done mostly sitting down), "somewhat active" (walking, playing with toys), and "very active" (activities that make a child "breathe hard and sweat," like running and skipping).

Skin pigmentation and UVB exposure - Skin color (type) for the infant will be established by taking the average of three measurements at each site for constitutive pigmentation at the inner upper arm and facultative pigmentation (UVB exposure) at the forehead, mid-forearm and lower leg using a spectrophotometer (CM-700d/600d, Konica Minolta, USA). Individual typological angle (ITA^{o}) will be calculated with the L* and b* values using published equations [10]. Using constitutive pigmentation, infants will be classified into skin types (I-III: white; IV-VI: non-white) based on Fitzpatrick descriptions [11, 12]. Sun exposure, winter travel and use of sun block will also be surveyed. Sun exposure is expressed as a percentage of body surface area (BSA) exposed and then sun index calculated for each child by multiplying the percent BSA exposed by the time spent outside (min/d); this index does not include sun block [13]. Season of visit will be defined by equinox/solstice [14].

3.10 Will health service research issues be addressed?

Justify inclusion/exclusion. At this time, we will not seek these analyses. The trial is in otherwise healthy infants who are not expected to seek health services for nutritional interventions beyond the regular physician visits for routine care. Supplements for infants are covered by the Quebec Health Insurance program based on family economic status. We will document any additional testing associated with the trial such as in the event of high ionized calcium. We will record any adverse events and general illnesses. Based on our previous dose-response trial [3], we have experience with this and will document any health care use outside of routine care. This includes types of tests, duration of investigations including hospital time, parent time incurred etc.

3.11 What is the proposed sample size and what is the justification?

The minimum sample size (n=66) is based on the primary objective, lean mass in g at 3 mo of age and an effect size of 9% (400 g), SD of 670 g, an allocation ratio of 1:1, power of 80 and alpha of 0.01. Accounting for possible dropouts (≤10%) or missing data (1% due to movement artifact) we will study 74 infants/group. The attrition is based on 3 similar studies with an average attrition of 9% [3, 15, 16]. We have used a more stringent estimate based on an alpha of

1% to enable multiple comparisons over time and in the event of greater variability in lean mass. We believe the estimated effect size is clinically meaningful since infants in the preliminary healthy status group treated with 400 IU/d increased lean mass between 1 mo and 3 mo by 670 g and the **Δ** 400 g anticipated between our trial groups by 3 mo represents 60% of that normal change which would be enough to reset growth for a leaner body phenotype.

3.12 What is the planned recruitment rate?

What evidence is there that it is achievable? We will recruit the 148 infants for the trial over the first 3 of 6 grant years (4 to 5 infants/month) as well as the reference group (n=74); also see **Table 2**. We will ensure the reference group is frequency matched by season to reflect the trial's proportion of white:non-white infants. The recruitment rates are realistic since in the past we have recruited 227 infants over 3.5 years for 3 different studies [3, 15, 16] related to vitamin D out of one hospital and 5 pediatric clinics. Assuming ~24% of infants have low vitamin D status, the estimate for screening is at least 592 infants. To account for those not wishing to participate we will screen 2000 neonates yearly. Given the recruiting hospitals, St. Justine Hospital which specialises in maternal-child health and where co-applicant Dr. Wei is appointed and actively recruits for trials [17]; McGill University Hospital Centre; and Lakeshore General Hospital where Dr. Weiler has recruited, we do not anticipate having difficulty meeting these numbers.

3.13 Are there likely to be any problems with compliance?

Based on our studies [3, 15, 16], compliance for vitamin D supplementation is very high (86-90%) in breast fed and weaned infants (84%). We will educate the parents on the importance of giving the supplement at all times regardless of intake of vitamin D from other sources (mother's milk, weaning milks, egg yolk etc). Compliance will be evaluated by parent self-report of dosages given as ascertained at each visit, by pre and post-weighing of bottles of supplement used and our biological response data (serum 25(OH)D).

3.14 What is the likely rate of loss to follow up?

We anticipate a 5-10% loss to follow-up. Previously we had <4% loss to follow-up at 3 mo [15]. In our dose-response study [3] a higher loss to follow-up of 12% was ascribed to additional blood sampling for safety assessments; which is not as likely in the present study design where we will use lower dosages (i.e. 1000 IU/d vs 1600 IU/d). We published our attrition data which were initially higher when the safety issues were being resolved and learned that healthy infants have higher ionized calcium than previously thought [6].

3.15 How many centers will be involved?

One research centre with recruitment from 1 hospital that we work with; recruitment site will be documented for each infant and considered in the analyses.

3.16 What is the proposed type of analyses?

Intent-to-treat analyses. All data entry will be audited and baseline characteristics expressed as mean (SD) or median (IQR) depending on normality for continuous data or as proportions for ordinal data (e.g. white non-white, sex). Continuous data for primary outcome measurements will be expressed as mean (95% CI) or proportion (95%CI). All data analysis will be conducted using SAS (version 9.3, SAS Inst. and SAS University Edition). Continuous data for the primary outcome will be examined using mixed model ANOVA accounting for fixed effects (sex,

dosage, time, dosage*time) and random effects (e.g. infant ID.) with post-hoc testing as applicable (i.e. 2 treatment groups and 1 reference group over 4 time-points) using Tukey-Krameer correction and significant differences accepted at $p<0.05$. The mixed model ANOVA does not require that data be parametric nor of homogeneous variance. Data will be tested using Kolmogorov-Smirnov, Anderson-Darling, or a Shapiro-Wilk tests for normality and where necessary transformed (e.g. log value etc.) to meet the assumptions of the post-hoc tests. The mixed model uses all available data, missing data does not result in omission of an infant's existing data. If more than 5% of data is missing, imputation approaches will be considered. Compliance (unused product and surveyed data) will be compared between groups. For the secondary objectives, a MIXED model will be used for continuous data. The proportion of those achieving the 50 and 75 nmol/L targets will be expressed as a proportion (95% CI) and differences among groups tested using Chi-square analyses or other similar tests based on numbers in each cell or logistic regression. We are experienced in all of these tests [3, 15]. The healthy reference group will be statistically analyzed to form reference curves (e.g., lean mass) using LMS methods [18].

3.17 What is the proposed frequency of analyses?

For the primary outcome, one analysis will be conducted on blinded groups after all infants have completed 12 mo of study and a second analysis again at 3 y. We wish to keep all of the staff blinded across the entire study. This can be accomplished by generating a database of participants and by removing dates of birth and dates of visit.

3.17 Are there any planned subgroup analyses?

For the primary objective we do not have subgroup analyses planned, but have considered possibilities. In the event that infant sex is not balanced we will use sex as a covariate. The potential subgroup analyses we have identified are measured skin color (pigmentation). These would only be explored in efforts to generate hypotheses and consequently to design future studies. Skin pigmentation is embedded in the design as infant skin color will be used as stratification criteria. Our objective measures of pigmentation could improve understanding the biological response to vitamin D.

4. Trial management

4.1 What are the arrangements for day to day management of the trial?

Refer to Table 2.

Randomization: Randomization will be set using RANDOMIZE.net which is a fee-for-service website. It enables randomization according to random block sizes, stratification, 2 treatment arms and verification of entry criteria; and will be used to track non-randomized infants (reference group).

Data handling: Two PhD trainees will be responsible for data entry including all screening data and data from visits. Data will be entered after each visit or as soon as when biochemistry data is available; our study co-ordinator (and registered nurse) will audit all of the data and communicate any abnormal findings to the study physicians ASAP. Any abnormal test will be repeated within 24 h and if still abnormal the child will move to standard of care (open label 400 IU/d) and be followed as intent-to- treat.

4.2 Describe the trial steering committee and if relevant the data safety and monitoring committee.

Since this trial uses vitamin D within amounts suitable for the general public we will not have a monitoring committee as required in the past [3]; see section 4.1 data handling above and **Diagram 2 and Table 3.** Each of the applicants will be a member of the steering committee and will meet prior to launching the study to review all procedures; then 6-monthly. The safety committee is comprised of a research nurse, the PI and 3 MDs who are co-applicants. We did not seek an arm's length safety officer since our dosages are ≤ to the IOM UL. Dr. Wei can also advise if maternal vitamin D status is low.

4.3 Describe any risks to the safety of participants involved in the trial.

The vitamin D dosages planned in this study are within the UL recommendations set for the general public by the IOM [19, 20] . We thus believe the risks associated with the interventions to be minimal. Nonetheless, we will monitor for status until 6 mo of age; the UL for vitamin D thereafter is higher at 1500 IU/d. The risks associated with blood sampling are minimized by small volumes. Lastly, dual-energy x-ray absorptiometry (DXA) scans are deemed minimal risk according to the International Society for Clinical Densitometry [21].

Diagram 1: Randomization scheme

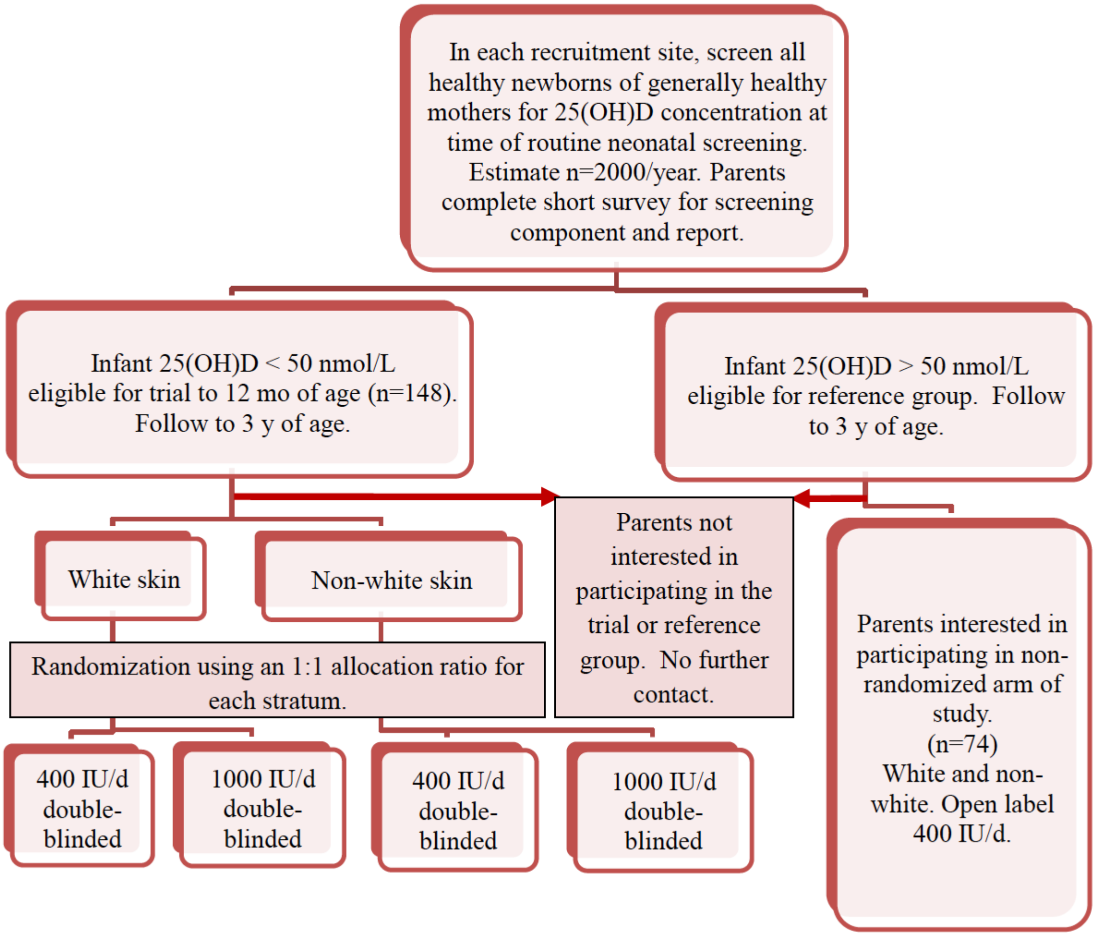

Diagram 2: Safety protocol - For use at inception 3, 6 and 12 months

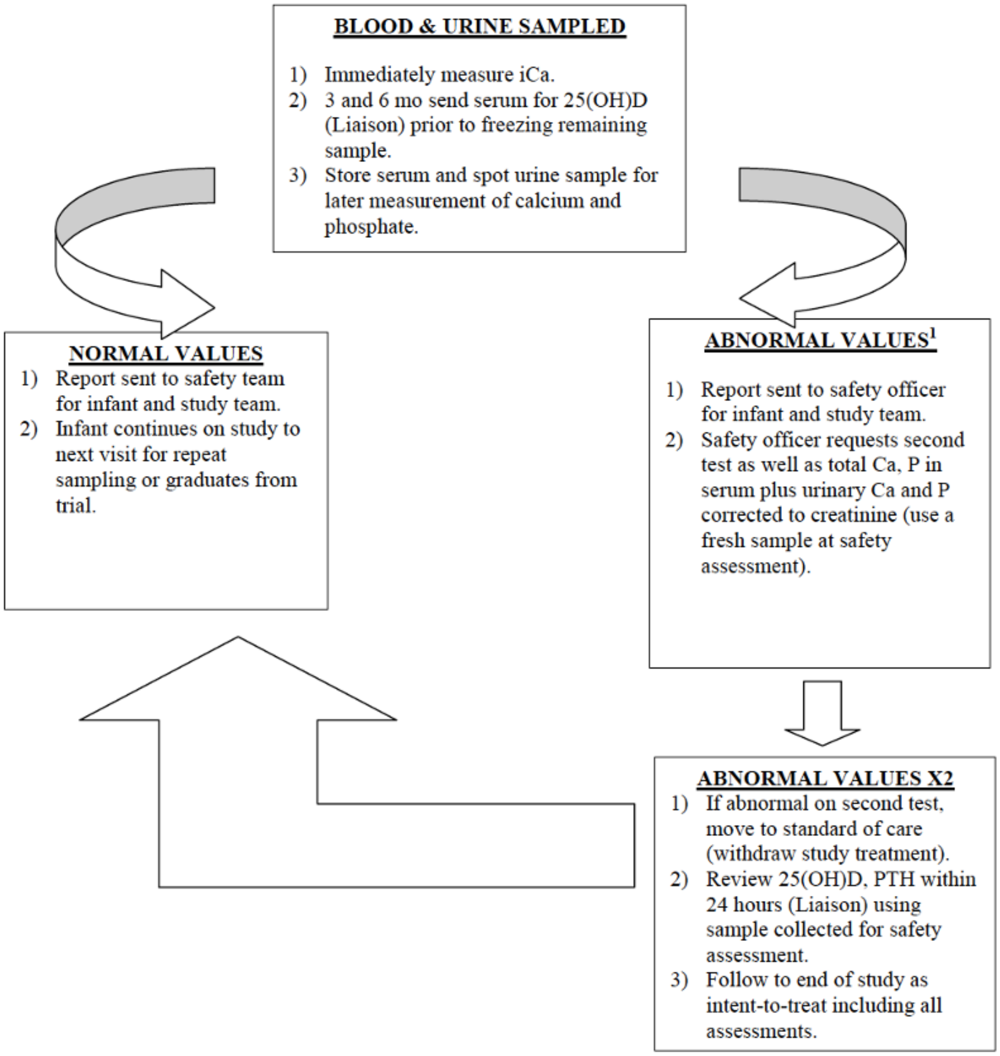

Additional Aspects of Safety Monitoring.

Should an infant be identified with growth failure, defined as crossing major percentiles a report will be sent to the infant's physician for follow-up. Since there are no standards by which to judge infant lean, fat and bone mass, changes in these will be examined. In any withdrawal of treatment scenario, these infants will continue on study unless advised otherwise by the physician and if treatment withdrawal is warranted, they will be followed as intent-to-treat.

Table 1. Summary of study measurements and timing

Measurement	Screening	Baseline	3 mo	6 mo	12 mo	24 & 36 mo
**Biochemistry**						
Total 25(OH)D by Liaison	x		x	x		
Serum vitamin D metabolites by LC-MS/MS<br>25(OH)D<br>C-3 alpha epimer of 25(OH)D<br>$24,25(OH)_2D$<br>$1,25(OH)_2D$		x	x	x	x	x
Parathyroid hormone		x	x	x	x	x
Ionized calcium, total Ca and PO_4		x	x	x	x	x
Urinary calcium:creatinine		x	x	x	x	
IGF-1 and IGFBP3		x	x	x	x	x
Epigenetic mechanisms		x	x			
**Anthropometry**						
Weight	x (chart)	x	x	x	x	x
Length (height)		x	x	x	x	x
Head Circumference		x	x	x	x	x
Body composition and bone by DXA		x	x	x	x	x
**Surveyed Information**						
Obstetrical record	x (chart)					
Demographic surveys	x	x				
Adverse events/any illnesses		x	x	x	x	x
Dietary/supplement intake & compliance		x	x	x	x	x
Sun exposure/skin pigmentation		x	x	x	x	x
Activity (HAES)						x

Abbreviations: DXA: dual-energy x-ray absorptiometry; HAES: habitual activity estimation scale; IGF-1: insulin like growth factor-1; IGFBP3: insulin like growth factor binding protein 3; LC-MS/MS: liquid chromatrography tandem mass spectroscopy.

Table 2. Study time-lines (6 year grant October 1, 2015 to September 30, 2021)

Period	Activities	Comments
July-October 2015	• Funding notice (July)<br>• Steering committee meeting (July)<br>• Ethics review (Aug)<br>• Students begin program (Sept)<br>• Register trial www.clinicaltrials.gov<br>• Set up RANDOMIZATION.net<br>• Printing of study materials<br>• Prepare/receive supplements	All staff are currently available to transfer from other projects.<br><br>Letter of no objection from Health Canada will be obtained.<br><br>Euro-Pharm has agreed to provide supplements in kind – see budget page attachments for letter of support.
October 2015-2018	Begin screening recruitment at each hospital's newborn unit (*Ethics review in Quebec hospitals is now streamlined so we apply to one site and the others adopt the certificate*):<br>• Ste. Justine Hospital<br>• Lakeshore General Hospital<br>• McGill University Health Centre (new 2015 Glen site)	Recruitment is to be overseen by Dr. Wei. Study staff and trainees will visit each hospital 5 d a week to recruit for screening. Parent(s) of healthy newborn infants born to generally healthy mothers will be asked if they would like vitamin D status measured as screening for a trial; and if so this would be done using blood taken at the same time as newborn screening. Short demographic and obstetric surveys would be completed. Vitamin D will be measured daily and parents contacted to see if they would like to participate in the trial depending on vitamin D status below 50 nmol/L 25(OH)D or above (reference group). Results of the screening will be given to parents; not thereafter unless safety issues arise.
October 2015-2018	Recruitment to trial and begin study sampling, measurements and surveys. Data entry is ongoing as collected.	Trial is held at Mary Emily Clinical Nutrition Research Unit of the School. All aspects conducted at this site as overseen by the PI; and safety monitoring communicated to study physicians by research nurse.
March 2016	Steering Committee meetings every 6 mo from this point.	Meetings mixed format; in person for those local and by joined teleconference for McNally and Jones. All investigators, staff, trainees.
October 2018	Complete and clean database on screening.	The screening data is valuable, the data can be written up as a report for presentation at scientific conferences and publishing in peer-review journals (open access).
January-March 2019	Epigenetics	Begin analyses using the animal study; then later in year 4 work with baseline and 3 mo buffy coat samples; extraction of DNA and methylation studies. Communicate results as these become available, scientific meetings and peer-review journals.
October 2019-	Complete biochemistry. Complete and clean database on trial.	Begin analysis of data and communicate results. Publish in peer-review journals (open-access format).
October 2017-2021	Complete 2-3 y data points including sampling, biochemical analyses, surveyed information etc. Build and clean database, reporting.	Communicate results, scientific meetings and peer-review journals.

Table 3. Normative values and threshold values used in safetymonitoring[1]

<table>
<tr><td>25(OH)D</td><td>Safe range <225 nmol/L according to Canadian Paediatric Society (CPS) and using our in-house Liaison total 25(OH)D (Diasorin) to enable 24 h assessments. This assay may overestimate by up to 20% (1) and thus we have used the CPS guidelines here for safety assessments only. None of the infants in the previous trial (2, 3) had values above this if vitamin D dosage was ≤1200 IU/d and using LC-MS/MS analyses. If value exceeds this limit then review plasma and urine mineral concentrations and consider moving to standard of care based on physician assessment; if these are normal, then repeat sample in 3 mo.</td></tr>
<tr><td>PTH</td><td>Normal range 10-60 pg/ml. If value <10 or >60, PTH to be repeated with plasma and urine minerals. As long as these are within the normal range, follow as per study routine. If any mineral concentrations are abnormal as per the criteria defined below.</td></tr>
<tr><td>Plasma Ca</td><td>Normal range 2.2 –2.6 mmol/L. If value <2.0 or >2.6, repeat sample and include ionized Ca, P, and urine minerals. If total Ca <2.0 and ionized Ca <1.0, break study code. If total Ca >2.6 and ionized Ca > age specific 95% percentile and urine Ca:Cr >2.2, safety officers unblind the infant, move to standard of care and send for urgent endocrine assessment.</td></tr>
<tr><td>Ionized Ca</td><td>Normal range for age using data from our previous trial (2, 3) which has been adopted by children's hospitals. If value is outside of 2.5th to 97.5th percentile range (see table below), sample to be repeated within 24 h. If repeated values are abnormal, safety officers to unblind that infant, move to standard of care or other appropriate action and potentially send for urgent endocrine assessment.
<table>
<tr><td>Percentile/Age</td><td>≤1 mo</td><td>3 mo</td><td>6 mo</td><td>12 mo</td></tr>
<tr><td>2.5th percentile</td><td>1.32</td><td>1.31</td><td>1.29</td><td>1.25</td></tr>
<tr><td>97.5th percentile</td><td>1.47</td><td>1.46</td><td>1.41</td><td>1.39</td></tr>
</table>
</td></tr>
<tr><td>Plasma phosphorus</td><td>Normal range for infants, 1.55-2.65 mmol/L. If <1.0 or >3.0, repeat with plasma Ca and urine mineral profile. If persistently abnormal, send for urgent endocrine/nephrology assessment.</td></tr>
<tr><td>Urine Ca: creatinine</td><td>Urine Ca:cr >2.2combined with total plasma Ca >2.6 and ionized plasma Ca >97.5th percentile, safety officers to unblind as above for plasma Ca.</td></tr>
<tr><td>Urine P: tubular reabsorption of phosphorus</td><td>Tubular reabsorption of phosphorus (TRP) normal: 85-100%. If concentration abnormal, verify with a repeat sample and assess serum mineral status. If plasma concentrations are normal, repeat in 3 months. If persistently low, unblind infant at this 3 month point.</td></tr>
</table>

[1] Based on values currently used at the Montreal Children's, Shriners, CHEO and General Hospitals.

5. References

1. Arbuckle TE, Wilkins R, Sherman GJ. Birth weight percentiles by gestational age in Canada. Obstet Gynecol. 1993;81(1):39-48. PubMed PMID: 8416459.
2. Rasmussen KM, Catalano PM, Yaktine AL. New guidelines for weight gain during pregnancy: what obstetrician/gynecologists should know. Curr Opin Obstet Gynecol. 2009 Dec;21(6):521-6. PubMed PMID: 19809317. Pubmed Central PMCID: 2847829.
3. Gallo S, Comeau K, Vanstone C, Agellon S, Sharma A, Jones G, et al. Effect of different dosages of oral vitamin D supplementation on vitamin D status in healthy, breastfed infants: a randomized trial. JAMA. 2013 May 1;309(17):1785-92. PubMed PMID: 23632722.
4. Kaufmann M, Gallagher JC, Peacock M, Schlingmann KP, Konrad M, DeLuca HF, et al. Clinical utility of simultaneous quantitation of 25-hydroxyvitamin D and 24,25-dihydroxyvitamin D by LC-MS/MS involving derivatization with DMEQ-TAD. J Clin Endocrinol Metab. 2014 Jul;99(7):2567-74. PubMed PMID: 24670084. Pubmed Central PMCID: 4079315.
5. Tabatabaei N, Rodd CJ, Kremer R, Khavandgar Z, Murshed M, Weiler HA. Dietary vitamin D during pregnancy has dose-dependent effects on long bone density and architecture in guinea pig offspring but not the sows. J Nutr. 2014 Dec;144(12):1985-93. PubMed PMID: 25320192.
6. Gallo S, Comeau K, Sharma A, Vanstone CA, Agellon S, Mitchell J, et al. Redefining normal bone and mineral clinical biochemistry reference intervals for healthy infants in Canada. Clin Biochem. 2014 Oct;47(15):27-32. PubMed PMID: 25064025.
7. Scanlon KS, Alexander MP, Serdula MK, Davis MK, Bowman BA. Assessment of infant feeding: the validity of measuring milk intake. Nutr Rev. 2002 Aug;60(8):235-51. PubMed PMID: 12199299. Epub 2002/08/30. eng.
8. Hay J. Development and testing of the Habitual Activity Estimation Scale. Eur J Phys Educ. 1997;2:110.
9. Hay J. Adequacy and predilection for activity in children. Clin J Sports Med. 1992;2:192-201.
10. Chardon A, Cretois I, Hourseau C. Skin colour typology and suntanning pathways. Int J Cosmet Sci. 1991 Aug;13(4):191-208. PubMed PMID: 19291061. Epub 1991/08/01. eng.
11. Del Bino S, Sok J, Bessac E, Bernerd F. Relationship between skin response to ultraviolet exposure and skin color type. Pigment Cell Res. 2006 Dec;19(6):606-14. PubMed PMID: 17083487. Epub 2006/11/07. eng.
12. Reeder AI, Hammond VA, Gray AR. Questionnaire items to assess skin color and erythemal sensitivity: Reliability, validity, and "the Dark Shift". Cancer Epidemiol Biomarkers Prev. 2010 May;19(5):1167-73. PubMed PMID: ISI:000278489800004.
13. Barger-Lux MJ, Heaney RP. Effects of above average summer sun exposure on serum 25-hydroxyvitamin D and calcium absorption. J Clin Endocrinol Metab. 2002 Nov;87(11):4952-6. PubMed PMID: 12414856.
14. Webb AR, Kline L, Holick MF. Influence of season and latitude on the cutaneous synthesis of vitamin D_3: exposure to winter sunlight in Boston and Edmonton will not promote vitamin D_3 synthesis in human skin. J Clin Endocrinol Metab. 1988;67(2):373-8. PubMed PMID: 2839537.

15. Gallo S, Phan A, Vanstone CA, Rodd C, Weiler HA. The change in plasma 25-hydroxyvitamin D did not differ between breast-fed infants that received a daily supplement of ergocalciferol or cholecalciferol for 3 months. J Nutr. 2013 Feb;143(2):148-53. PubMed PMID: 23256143.
16. Rodd C, Jean-Philippe S, Vanstone C, Weiler H. Comparison of 2 vitamin D supplementation modalities in newborns: adherence and preference. Appl Physiol Nutr Metab. 2011 Jun;36(3):414-8. PubMed PMID: 21574782.
17. Wei SQ, Audibert F, Hidiroglou N, Sarafin K, Julien P, Wu Y, et al. Longitudinal vitamin D status in pregnancy and the risk of pre-eclampsia. BJOG. 2012 Jun;119(7):832-9. PubMed PMID: 22462640.
18. Gallo S, Vanstone CA, Weiler HA. Normative data for bone mass in healthy term infants from birth to 1 year of age. J Osteoporos. 2012;2012:672403. PubMed PMID: 23091773. Pubmed Central PMCID: 3468026.
19. Ross AC, Manson JE, Abrams SA, Aloia JF, Brannon PM, Clinton SK, et al. The 2011 Dietary Reference Intakes for Calcium and Vitamin D: what dietetics practitioners need to know. J Am Diet Assoc. 2011 Apr;111(4):524-7. PubMed PMID: 21443983.
20. Ross AC, Institute of Medicine (U.S.). Committee to Review Dietary Reference Intakes for Vitamin D and Calcium. Dietary reference intakes : calcium vitamin D. Washington, D.C.: National Academies Press; 2011. xv, 1115 p. p.
21. Kalkwarf HJ, Abrams SA, DiMeglio LA, Koo WW, Specker BL, Weiler H. Bone densitometry in infants and young children: the 2013 ISCD Pediatric Official Positions. J Clin Densitom. 2014 Apr-Jun;17(2):243-57. PubMed PMID: 24674638.

6. References for Safety Monitoring

1. Gallo S, Phan A, Vanstone CA, Rodd C, Weiler HA. The change in plasma 25-hydroxyvitamin D did not differ between breast-fed infants that received a daily supplement of ergocalciferol or cholecalciferol for 3 months. J Nutr. 2013 Feb;143(2):148-53. PubMed PMID: 23256143.
2. Gallo S, Comeau K, Sharma A, Vanstone CA, Agellon S, Mitchell J, et al. Redefining normal bone and mineral clinical biochemistry reference intervals for healthy infants in Canada. Clin Biochem. 2014 Oct;47(15):27-32. PubMed PMID: 25064025.
3. Gallo S, Comeau K, Vanstone C, Agellon S, Sharma A, Jones G, et al. Effect of different dosages of oral vitamin D supplementation on vitamin D status in healthy, breastfed infants: a randomized trial. JAMA. 2013 May 1;309(17):1785-92. PubMed PMID: 23632722
