# Supplementary material for: Effect of Vitamin D Supplementation on Bone Mass in Infants With 25-Hydroxyvitamin D Concentrations Less Than 50 nmol/L: A Prespecified Secondary Analysis of a Randomized Clinical Trial
Source: JAMA Pediatr. 2023 Feb 13;177(4):353–62. doi: 10.1001/jamapediatrics.2022.5837 (PMC9926359; doi:10.1001/jamapediatrics.2022.5837)
Supplement: Supplement 2. — eTable 1. Infant Breastfeeding Status and Age of Introduction of Solid Foods and Maternal Nutrient Intake From Food and Supplements During Pregnancy by Group eTable 2. Whole Body and Lumbar Spine Bone Mass of Infants by Groups Over Time eTable 3. Serum Vitamin D Metabolites of Infants by Groups Over Time eTable 4. Biomarkers of Calcium and Bone Metabolism of Infants by Groups Over Time eFigure 1. Whole Body BMC and Lumbar Spine BMC Accretion Rates of infant Groups Over Time eFigure 2. Proportions of Vitamin D Sufficient Infants by Groups Over Time [file jamapediatr-e225837-s002.pdf]

Supplemental Online Content

Gharibeh N, Razaghi M, Vanstone CA, et al. Effect of vitamin D supplementation on bone mass in infants with 25-hydroxyvitamin D concentrations less than 50 nmol/L: a prespecified secondary analysis of a randomized clinical trial. *JAMA Pediatr*. Published online February 13, 2023. doi:10.1001/jamapediatrics.2022.5837

eTable 1. Infant Breastfeeding Status and Age of Introduction of Solid Foods and Maternal Nutrient Intake from Food and Supplements During Pregnancy by Group
eTable 2. Whole Body and Lumbar Spine Bone Mass of Infants by Groups Over Time
eTable 3. Serum Vitamin D Metabolites of Infants by Groups Over Time
eTable 4. Biomarkers of Calcium and Bone Metabolism of Infants by Groups Over Time
eFigure 1. Whole Body BMC and Lumbar Spine BMC Accretion Rates of Infant Groups Over Time
eFigure 2. Proportions of Vitamin D Sufficient Infants by Groups Over Time

This supplementary material has been provided by the authors to give readers additional information about their work.

© 2023 Gharibeh N et al. *JAMA Pediatrics*.

eTable 1. Infant Breastfeeding Status and Age of Introduction of Solid Foods and Maternal Nutrient Intake from Food and Supplements During Pregnancy by Group

	400 IU/d trial	1000 IU/d trial	p-value[a]	Reference
**Infant**				
Breastfeeding status[b]				
1 mo	49/49 (100%)	49/49 (100%)	1.00	41/41 (100%)
3 mo	41/44 (93.2%)	41/44 (93.2%)	1.00	30/35 (85.7%)
6 mo	34/40 (85.0 %)	36/43 (83.7%)	.87	27/34 (79.4%)
12 mo	16/36 (44.4%)	17/42 (40.5%)	.72	12/32 (37.5%)
Age at introduction of solid foods (mo)	5.0 [0.9]<br>(range: 3.0 to 7.0)<br>(n=40)	4.9 [0.9]<br>(range: 3.0 to 6.0)<br>(n=43)	.41	4.9 [0.7]<br>(range: 4.0 to 7.0)<br>(n=33)
**Mother**[c]	(n=43)	(n=44)		(n=36)
Energy (kcal/d)	2137 [777]<br>1892 [1604-2586]	2191 [982]<br>1955 [1555-2634]		2091 [697]<br>1914 [1677-2446]
Protein (g/d)	92 [35]<br>87 [68-114]	98 [44]<br>85 (73-114)		96 [36]<br>89 (69-124)
Carbohydrates (g/d)	291 [130]<br>266 [212-343]	286 [147]<br>249 [181-343]		261 [83]<br>254 [205-327]
Fat (g/d)	73 [29]<br>63 [53-97]	78 [33]<br>75 [54-88]		79 [32]<br>72 [58-96]
Vitamin D (IU/d)	552 [222]<br>578 [388-676]	610 [241]<br>589 [491-774]		730 [289]<br>762 [552-915]
Calcium (mg/d)	1318 [444]<br>1250 [1014-1677]	1350 [554]<br>1304 [903-1677]		1500 [529]<br>1317 [1098-1780]
Magnesium (mg/d)	475 [161]<br>419 [355-606]	478 [173]<br>441 [350-598]		491 [119]<br>482 [407-581]
Phosphorus (mg/d)	1512 [547]<br>1411 [1148-1798]	1556 [700]<br>1363 [1089-1894]		1548 [577]<br>1405 [1129-2022]

[a]Differences between the trial groups in infant breastfeeding status and age of introduction of solid foods were tested using linear mixed-effects regression model (continuous variables) and Chi-square or Fisher's exact test (categorical variables). Abbreviations: IU: international units.
[b]Breastfeeding status (any type: exclusive or mixed) is reported as proportions (%) and age of introduction of solid foods as mean [SD] (minimum-maximum) (n).
[c]Data are reported as mean [SD] and median [IQR] for maternal intake during pregnancy with no hypothesis testing reported given that these characteristics consist of baseline covariates.

© 2023 Gharibeh N et al. *JAMA Pediatrics*.

eTable 2. Whole Body and Lumbar Spine Bone Mass of Infants by Groups Over Time

Variable[a]	400 IU/d trial	1000 IU/d trial	1000 IU/d minus 400 IU/d Difference (95%CI)	Group-by-time p-value	Reference
Whole body BMC (g)				.43	
1 mo	95.47 [13.03] (49/49)	95.81 [13.50] (49/49)	--		92.24 [15.85] (39/41)
3 mo	149.13 [20.85] (43/44)	150.46 [23.35] (44/44)	1.02 [-10.27, 12.32]		154.22 [24.61] (34/35)
6 mo	193.96 [24.93] (39/40)	194.29 [32.03] (41/43)	1.50 [-10.20, 13.20]		197.33 [31.90] (34/34)
12 mo	268.70 [39.71] (30/36)	278.63 [44.63] (37/42)	10.78 [-1.79, 23.34]		274.43 [39.76] (30/32)
Whole body BMC/weight (g/kg)				.70	
1 mo	24.4 [3.0] (49/49)	24.8 [2.4] (49/49)	--		24.2 [3.0] (39/41)
3 mo	23.7 [2.6] (43/44)	23.9 [2.6] (44/44)	0.2 [-0.9, 1.2]		24.7 [2.5] (34/35)
6 mo	24.9 [1.7] (39/40)	24.5 [2.6] (41/43)	-0.4 [-1.5, 0.7]		25.2 [3.2] (34/34)
12 mo	27.5 [2.4] (30/36)	27.8 [2.7] (37/42)	0.3 [-0.9, 1.5]		27.9 [2.8] (30/32)
Whole body BMC/length (g/cm)				.53	
1 mo	1.8 [0.2] (49/49)	1.8 [0.2] (49/49)	--		1.8 [0.3] (39/41)
3 mo	2.4 [0.3] (43/44)	2.4 [0.3] (44/44)	0.0 [-0.2, 0.1]		2.5 [0.3] (34/35)
6 mo	2.9 [0.3] (39/40)	2.8 [0.4] (41/43)	0.0 [-0.2, 0.1]		2.9 [0.4] (34/34)
12 mo	3.5 [0.4] (30/36)	3.6 [0.5] (37/42)	0.1 [-0.1, 0.3]		3.6 [0.5] (30/32)
Lumbar spine 1-4 BMC (g)				.83	
1 mo	2.11 [0.44] (48/49)	2.24 [0.38] (49/49)	--		2.20 [0.39] (40/41)
3 mo	2.55 [0.42] (44/44)	2.60 [0.44] (44/44)	0.07 [-0.18, 0.31]		2.71 [0.44] (35/35)
6 mo	3.22 [0.60] (40/40)	3.25 [0.53] (43/43)	0.05 [-0.20, 0.31]		3.39 [0.47] (34/34)
12 mo	4.97 [1.02] (34/36)	4.99 [0.74] (41/42)	0.09 [-0.17, 0.36]		4.96 [0.73] (32/32)
Lumbar spine 1-4 BMD (g/cm^2)				.11	
1 mo	0.214 [0.040] (48/49)	0.233 [0.046] (49/49)	--		0.225 [0.041] (40/41)
3 mo	0.211 [0.032] (44/44)	0.210 [0.029] (44/44)	0.001 [-0.015, 0.018]		0.223 [0.034] (35/35)
6 mo	0.232 [0.039] (40/40)	0.230 [0.034] (43/43)	0.000 [-0.016, 0.017]		0.248 [0.026] (34/34)
12 mo	0.296 [0.050] (34/36)	0.298 [0.035] (41/42)	0.008 [-0.010, 0.025]		0.303 [0.038] (32/32)

[a]Data are reported as mean [SD] (proportion of infants with useable scan).
Differences between the trial groups were tested using a linear mixed-effects regression model for group-by-time interaction and time; the model included participant-level random intercepts and slopes for time; post hoc testing showed no differences between trial groups over time (p>.05), with time (p<.001). Abbreviations: BMC: bone mineral content, BMD: bone mineral density, IU/d: international units per day, mo: months.

© 2023 Gharibeh N et al. *JAMA Pediatrics*.

eTable 3. Serum Vitamin D Metabolites of Infants by Groups Over Time

Variable[a]	400 IU/d trial	1000 IU/d trial	1000 IU/d minus 400 IU/d Difference (95%CI)	Group-by-time p-value	Reference
Serum 25(OH)D_3 (nmol/L)				<.001	
1	44.3 [14.3] (49/49)	44.7 [15.3] (48/49)	--		59.5 [15.3] (41/41)
3	77.4 [23.3] (44/44)	115.2 [35.3]* (43/44)	37.4 [27.4, 47.4]		89.8 [27.1] (35/35)
6	85.1 [18.6] (39/40)	121.6 [34.4]* (43/43)	37.9 [27.6, 48.2]		90.1 [28.5] (34/34)
12	82.3 [14.3] (36/36)	99.6 [28.8]# (42/42)	18.0 [7.4, 28.6]		94.7 [19.5] (31/32)
Serum 24,25$(OH)_2D_3$ (nmol/L)				<.001	
1	2.1 [1.3] (49/49)	2.0 [1.0] (48/49)	--		3.9 [3.9] (41/41)
3	4.5 [2.2] (44/44)	9.2 [4.8]* (43/44)	4.6 [3.5, 5.8]		5.3 [2.4] (35/35)
6	4.6 [2.1] (39/40)	8.8 [4.6]* (43/43)	4.1 [2.9, 5.4]		4.7 [2.3] (34/34)
12	4.0 [1.6] (36/36)	5.9 [3.3]# (42/42)	2.1 [0.8, 3.3]		4.4 [1.7] (31/32)
Ratio 25(OH)D_3:24,25$(OH)_2D_3$				<0.01	
1	26.3 [10.8] (49/49)	25.4 [9.9] (48/49)	--		18.9 [6.4] (41/41)
3	18.8 [5.9] (44/44)	13.8 [4.9]# (43/44)	-5.4 [-8.6, -2.3]		17.9 [4.8] (35/35)
6	19.3 [6.0] (39/40)	15.5 [5.5] (43/43)	-4.2 [-7.5, -1.0]		20.1 [5.0] (34/34)
12	21.7 [5.9] (36/36)	18.6 [5.4] (42/42)	-3.6 [-7.0, -0.3]		22.8 [7.0] (31/32)
Serum 3-epi-25(OH)D_3 (nmol/L)				<.001	
1	8.9 [5.6] (49/49)	9.9 [7.1] (49/49)	--		10.8 [5.2] (41/41)
3	13.6 [7.8] (44/44)	33.4 [25.0]* (43/44)	19.6 [15.0, 24.2]		14.8 [7.7] (35/35)
6	9.1 [5.7] (39/40)	19.4 [12.6]* (43/43)	10.2 [5.5, 15.0]		10.7 [5.3] (34/34)
12	6.2 [2.2] (36/36)	8.2 [4.0] (42/42)	2.2 [-2.7, 7.1]		6.4 [2.1] (31/32)

© 2023 Gharibeh N et al. *JAMA Pediatrics*.

Variable[a]	400 IU/d trial	1000 IU/d trial	1000 IU/d minus 400 IU/d Difference (95%CI)	Group-by-time p-value	Reference
Serum 1,25$(OH)_2D_3$ (pmol/L)				.55	
1	187.1 [74.3] (41/49)	160.3 [71.1] (41/49)	--		163.0 [72.6] (32/41)
3	185.3 [61.8] (39/44)	181.2 [58.2] (39/44)	-6.9 [-38.0, 24.2]		178.5 [59.6] (33/35)
6	184.9 [87.6] (38/40)	184.9 [69.1] (42/43)	-2.5 [-33.5, 28.5]		179.3 [63.2] (32/34)
12	204.2 [50.1] (32/36)	199.8 [82.4] (39/42)	-11.1 [-44.0, 21.9]		198.0 [53.0] (30/32)
Serum 1,24,25$(OH)_3D_3$ (pmol/L)				<.001	
1	30.6 [14.3] (41/49)	34.5 [16.0] (41/49)	--		39.8 [16.2] (32/41)
3	43.0 [14.4] (39/44)	69.2 [26.1]* (38/44)	25.5 [17.2, 33.7]		46.0 [15.9] (33/35)
6	39.8 [12.3] (38/40)	59.2 [26.5]* (43/43)	19.6 [11.5, 27.8]		39.5 [25.8] (32/34)
12	42.3 [14.0] (33/36)	49.6 [20.4] (41/42)	8.1 [-0.4, 16.7]		38.7 [14.8] (30/32)
Ratio 1,25$(OH)_2D_3$:1,24,25$(OH)_3D_3$				<.01	
1	8.9 [10.6] (41/49)	5.4 [2.3] (41/49)	--		4.6 [2.3] (32/41)
3	4.9 [2.4] (39/44)	3.0 [1.6] (38/44)	-1.9 [-3.9, 0.2]		4.3 [1.7] (33/35)
6	5.3 [4.3] (38/40)	3.7 [1.9] (42/43)	-1.6 [-3.6, 0.4]		5.8 [3.8] (32/34)
12	5.3 [2.3] (32/36)	4.5 [2.1] (39/42)	-1.1 [-3.3, 1.0]		5.8 [2.6] (30/32)

[a]Data are reported as mean [SD] (proportion of infants with sufficiently available serum). Differences between the trial groups were tested using a linear mixed-effects regression model for group-by-time interaction and time; the model included participant-level random intercepts and slopes for time; *p<.001, #p<.05 between trial groups, with time p<.05. Abbreviations: 25$(OH)D_3$: 25-hydroxyvitamin D_3, 1,25$(OH)_2D_3$: 1,25-dihydroxyvitamin D_3, 24,25$(OH)_2D_3$: 24,25-dihydroxyvitamin D_3, 1,24,25$(OH)_3D_3$: 1,24,25-trihydroxyvitamin D_3IU/d: international units per day, mo: months.

© 2023 Gharibeh N et al. *JAMA Pediatrics*.

eTable 4. Biomarkers of Calcium and Bone Metabolism of Infants by Groups Over Time

Variable[a]	400 IU/d trial	1000 IU/d trial	1000 IU/d minus 400 IU/d Difference (95%CI)	Group-by-time p-value	Reference
Urinary CTX-I:cr (mg/mmol)				.85	
1	66.7 [29.5] (49/49)	66.4 [34.9] (48/49)	--		55.9 [30.0] (41/41)
3	67.5 [25.7] (43/44)	68.3 [23.4] (44/44)	0.2 [-10.4, 10.9]		62.0 [(26.6] (35/35)
6	53.0 [23.4] (40/40)	47.2 [21.7] (42/43)	-6.4 [-17.3, 4.6]		56.2 [21.2] (33/34)
12	42.8 [17.1] (34/36)	42.1 [16.3] (41/42)	-1.3 [-12.7, 10.2]		37.2 [17.1] (32/32)
Plasma P1NP (ng/ml)				.64	
1	493.7 [168.0] (48/49)	522.5 [165.0] (48/49)	--		568.9 [558.7] (41/41)
3	568.1 [216.6] (43/44)	541.2 [248.2] (43/44)	-25.1 [-573.6, 523.5]		621.5 [185.3] (35/35)
6	616.4 [229.5] (40/40)	698.3 [464.7] (43/43)	80.1 [-479.1, 639.4]		662.7 [281.5] (34/34)
12	1269.6 [1982.1] (36/36)	1738.1 [3223.8] (39/42)	469.6 [-118.6, 1057.8]		1337.5 [2206.3] (32/32)
Plasma PTH (pg/ml)				.17	
1	38.8 [27.2] (48/49)	30.2 [19.1] (48/49)	--		31.7 [19.9] (41/41)
3	39.6 [23.5] (43/44)	30.8 [15.5] (43/44)	-9.0 [-18.2, 0.2]		30.0 [13.7] (35/35)
6	48.2 [26.7] (40/40)	38.6 [16.5] (43/43)	-9.7 [-19.1, -0.2]		38.9 [15.3] (34/34)
12	48.2 [24.8] (36/36)	47.6 [17.7] (39/42)	-2.7 [-12.5, 7.1]		46.7 [19.5] (32/32)
Blood iCa (mg/dL)				.49	
1	5.77 [0.24] (49/49)	5.81 [0.24] (49/49)	--		5.81 [0.16] (40/41)
3	5.69 [0.012 (44/44)	5.65 [0.16] (44/44)	-0.04 [-0.12, 0.08]		5.65 [0.40] (34/35)
6	5.53 [0.40] (40/40)	5.53 [0.12] (41/43)	0.00 [-0.08, 0.12]		5.53 [0.12] (33/34)
12	5.37 [0.20] (35/36)	5.45 [0.20] (42/42)	0.08 [-0.04, 0.16]		5.45 [0.16] (32/32)

© 2023 Gharibeh N et al. *JAMA Pediatrics*.

Variable[a]	400 IU/d trial	1000 IU/d trial	1000 IU/d minus 400 IU/d Difference (95%CI)	Group-by-time p-value	Reference
Urinary calcium:cr (mol:mol)				.58	
1	1.40 [1.00] (49/49)	1.30 [0.80] (48/49)	--		1.70 [1.20] (41/41)
3	1.50 [1.00] (43/44)	1.70 [1.00] (44/44)	0.13 [-0.21, 0.48]		1.40 [0.90] (35/35)
6	1.20 [0.80] (40/40)	1.10 [0.70] (42/43)	-0.13 [-0.48, 0.22]		1.20 [0.60] (33/34)
12	0.60 [0.50] (34/36)	0.70 [0.60] (41/42)	0.09 [-0.28, 0.46]		0.60 [0.40] (32/32)
Urinary phosphate:cr (mol:mol)				.97	
1	1.5 [1.5] (49/49)	1.5 [2.4] (48/49)	--		1.4 [1.2] (41/41)
3	1.4 [1.6] (43/44)	2.3 [5.6] (44/44)	0.94 [-2.76, 4.66]		2.2 [3.2] (35/35)
6	6.1 [7.2] (40/40)	6.8 [8.6] (43/43)	0.82 [-3.00, 4.63]		3.8 [4.2] (33/34)
12	18.7 [15.5] (34/36)	18.2 [16.5] (41/42)	-0.52 [-4.53, 3.50]		17.5 [12.9] (32/32)

[a]Data are reported as mean [SD] (proportion of infants with sufficiently available sample volume). Differences between the trial groups were tested using a linear mixed-effects regression model for group-by-time interaction and time; the model included participant-level random intercepts and slopes for time; post hoc tests showed no differences between trial groups over time (p>.05), with time p<.001. Abbreviations: CTX-I: alpha telopeptide of Type-1 collagen, P1NP: procollagen Type-1 N-terminal propeptide, PTH: parathyroid hormone, IU/d: international units per day, mo: months.

© 2023 Gharibeh N et al. *JAMA Pediatrics*.

eFigure 1. Whole Body BMC and Lumbar Spine BMC Accretion Rates of infant Groups Over Time

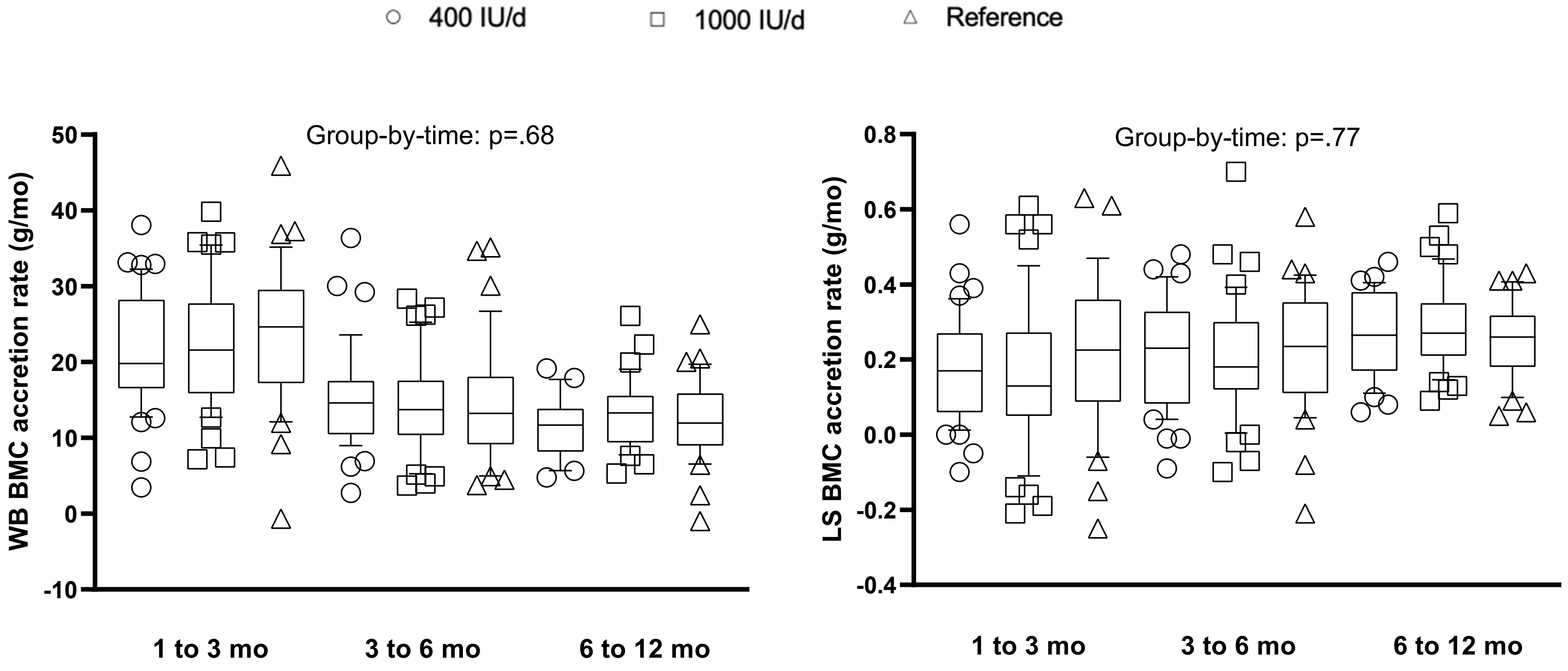

Data are reported using box-and-whiskers plots. The box extends from the 25th to 75th percentiles. The line in the middle of the box is plotted at the median. The whiskers are drawn down to the 10th percentile and up to the 90th. Points below and above the whiskers are drawn as individual dots. Data were analyzed using a linear mixed-effect model accounting for group-by-time interaction, and time; the model included participant-level random intercepts and slopes for time (p<.05, post hoc adjustment). Abbreviations: BMC: bone mineral content, BMD: bone mineral density, IU: international units.

© 2023 Gharibeh N et al. *JAMA Pediatrics*.

eFigure 2. Proportions of Vitamin D Sufficient Infants by Groups Over Time

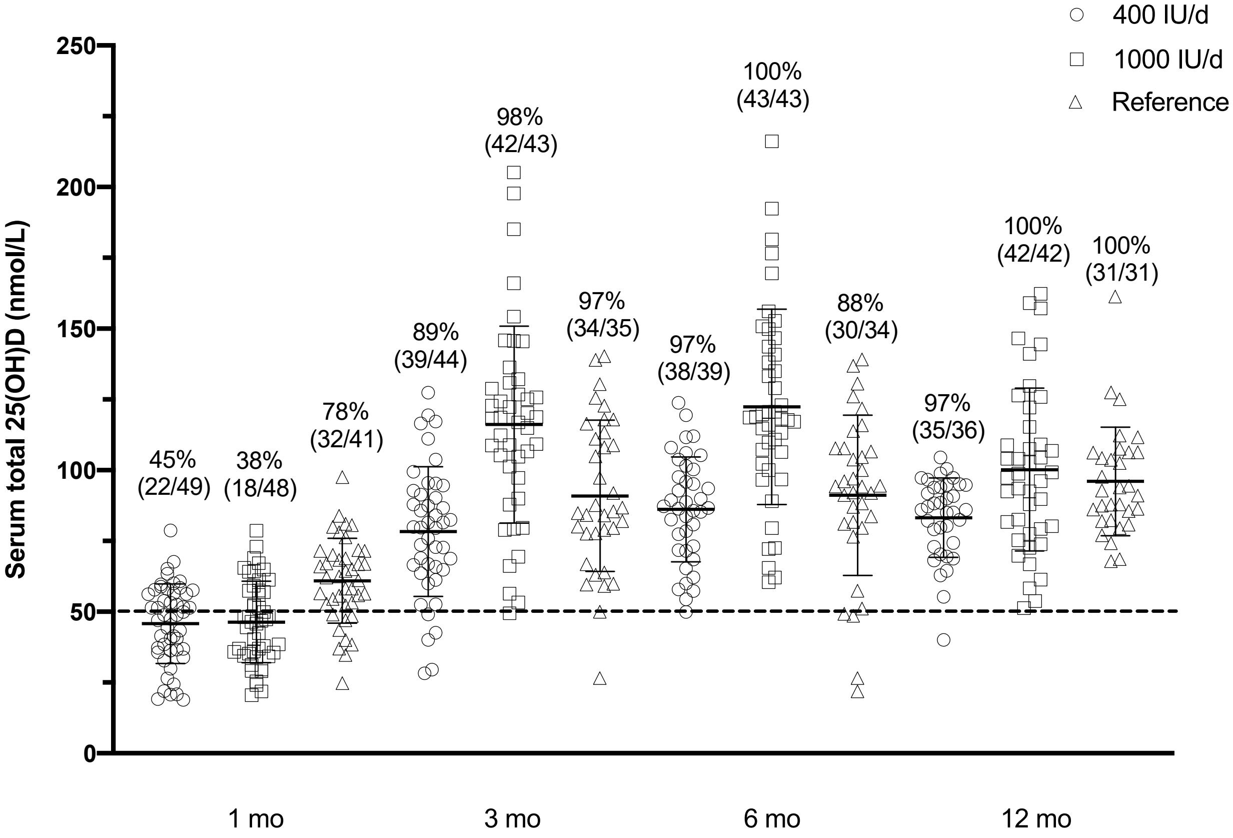

Data are reported as mean [SD]. The dashed line shows the cut-point for sufficiency (25(OH)D ≥50 nmol/L). Differences in proportions of vitamin D sufficient infants over time (3 to 12 months) among the trial groups were tested using the proc glimmix function, the CHISQ option in the model statement, and the effects of group-by-time interaction and time. Random effect of the participant was accounted for in the model. Proportions of vitamin D sufficient infants were not different among groups at 3, 6, and 12 months timepoints (p>.05). Abbreviations: 25(OH)D: 25-hydroxyvitamin D, and IU: international units.

© 2023 Gharibeh N et al. *JAMA Pediatrics*.
