# Supplementary material for: Effect of Vitamin D Supplementation on Bone Mass in Infants With 25-Hydroxyvitamin D Concentrations Less Than 50 nmol/L: A Prespecified Secondary Analysis of a Randomized Clinical Trial
Source: JAMA Pediatr. 2023 Feb 13;177(4):353–62. doi: 10.1001/jamapediatrics.2022.5837 (PMC9926359; doi:10.1001/jamapediatrics.2022.5837)
Supplement: Supplement 3. — Data Sharing Statement [file jamapediatr-e225837-s003.pdf]

Data Sharing Statement

Gharibeh. Effect of Vitamin D Supplementation on Bone Mass in Infants With 25-Hydroxyvitamin D Concentrations Less Than 50 nmol/L. *JAMA Pediatr*. Published February 13, 2023. doi:10.1001/jamapediatrics.2022.5837

Data
Data available: No

Additional Information
Explanation for why data not available: The data described in the manuscript will not be made available because permission to share data was not requested at the time of obtaining participant consent.
